# Global Burden of Inflammatory Bowel Disease Among Children and Adolescents: A Comprehensive Analysis (1990–2019)

**DOI:** 10.3389/ijph.2024.1607440

**Published:** 2024-09-09

**Authors:** Xuejie Chen, Xin Xiang, Xiaofei Fan, Weitong Xia, Yi Xiao, Sidan Wang, Shuyu Ye, Meng Kang, Fangmin Jing, Xing Wu, Yang Chen

**Affiliations:** ^1^ Department of Gastroenterology, Third Xiangya Hospital of Central South University, Changsha, Hunan, China; ^2^ Department of Clinical Medicine, Shandong Medical College, Jinan, Shandong, China; ^3^ Nursing Department, Third Xiangya Hospital of Central South University, Changsha, Hunan, China

**Keywords:** mortality, trends, inflammatory bowel disease, global burden of disease, early-onset

## Abstract

**Objective:**

We summarize the global, regional, and national burden of inflammatory bowel disease (IBD) in children and adolescents from 1990 to 2019.

**Methods:**

Based on the Global Burden of Disease Study 2019, the data of IBD in children and adolescents were analyzed by sex, age, year, and location. Joinpoint analysis was applied to assess the temporal trend of the disease burden.

**Results:**

From 1990 to 2019, the incidence of IBD in children and adolescents increased by 22.8%, from 20,897.42 to 25,658.55 cases, especially in high SDI region. During the same period, the DALY numbers decreased by 53.5%, from 243,081.06 to 113,119.86, with all SDI regions experiencing a clear drop in DALYs except high SDI regions. In 2019, early-onset IBD incidence and DALY numbers were reported at 2,053.52 (95% UI: 1,575.62 to 2,677.49) and 73,797.46 (95% UI: 43,655.86 to 105,998.63), respectively.

**Conclusion:**

Early-onset IBD in children and adolescents remains a significant global health concern. The disease burden has not improved in developed countries over the past 30 years, highlighting the need for targeted interventions.

## Introduction

Inflammatory bowel disease (IBD) encompasses a group of chronic intestinal disorders of unknown cause, characterized by non-specific inflammation, including conditions such as ulcerative colitis (UC) and Crohn’s disease (CD) [1]. The global surge in incidence rates has elevated childhood-onset IBD to a top concern among pediatric gastroenterologists, around 25% of IBD patients first exhibit symptoms before the age of 20 [2]. Among various factors such as genetics, environment, diet and lifestyle, infections, and immunological factors that associated with IBD onset, genetics may play a more significant role in the development of IBD in children and adolescents compared to adults [3–6]. Furthermore, factors such as cesarean delivery, limited exposure to breast milk, high dietary fat intake, and early antibiotic exposure have been identified as potential risk factors for the development of IBD in children and adolescents [7–10]. Studies have shown familial involvement in 20% of children with IBD, and heritability is greater with earlier onset in IBD [6, 11, 12].

Despite the current lack of comprehensive epidemiological studies on pediatric and adolescent IBD, it is undeniable that the burden of IBD among children and adolescents worldwide are on the rise [7, 13]. Some research indicates that in the United States and Canada, the incidence rate of IBD among children is approximately 10 cases per 100,000 children, with a continuing upward trend. Specifically, between 1996 and 2006, the annual incidence rate per 100,000 individuals for CD escalated from 2.2 to 4.3, while for UC, it rose from 1.8 to 4.9 [14, 15]. Compared to IBD in adults, the disease burden of IBD in children and adolescents is more severe, especially early-onset IBD [16]. Owing to the chronic disease progression with currently no cure, IBD not only causes physical, but also mental health, and educational problems for children and adolescents [12]. Due to the unique considerations for children with IBD, the treatment goals for pediatric IBD should focus on controlling symptoms, restoring growth, and preventing delays in puberty [12, 17–19]. This necessitates a different therapeutic approach for pediatric IBD compared to adult IBD. Current research suggests that IBD onset during childhood may represent a more severe symptom than those diagnosed in adults, requiring a higher level of treatment and care [20, 21].

Grasping the overall incidence and trends of IBD among children and adolescents across various global regions is pivotal for etiological studies of IBD and for the strategic distribution of healthcare resources. Our analysis utilized data from the Global Burden of Disease (GBD) study spanning from 1990 to 2019, aiming to delineate the burden of IBD in individuals under 20 years of age on a global scale, across different regions, socio-demographic index (SDI) categories, and countries.

## Methods

### Date Source

The GBD 2019 study provided estimates for incidence, mortality, and disability-adjusted life year (DALY) linked to 369 diseases and injuries across 204 countries and territories, covering the period from 1990 to 2019 [22]. We retrieved data on the incidence, prevalence, mortality, and DALY of IBD among children and adolescents spanning from 1990 to 2019 across 204 countries and regions (http://ghdx.healthdata.org/gbd-results-tool).

### Sociodemographic Index

The SDI is a composite metric assessing a country’s level of sociodemographic development. It incorporates factors such as *per capita* income, educational attainment, and the total fertility rate [23]. The SDI scale ranges from 0 to 1, where 0 signifies the lowest levels of *per capita* income, and 1 denotes the highest levels of income and education. Utilizing the SDI values for 2019, countries and regions were categorized into five distinct groups: low, low-middle, middle, high-middle, and high SDI.

### Joinpoint Poisson Regression

The temporal trends of IBD among children and adolescents were assessed using the Joinpoint Regression Model, a series of statistically linear models. Annual percent change (APC) with 95% CI for the entire period was reported. Joinpoint model identified the best fit for joinpoints at which an apparent change in trend is statistically significant. A maximum number of two joinpoints were allowed, and a Monte Carlo Permutation method was used for model selection. If the APC was significantly different from 0, an increasing (worsening) or decreasing (improving) trend was defined; if there was no difference from zero noted, a stable or level trend was defined [23]. The annual percentage changes (APCs) along with their 95% confidence intervals (CIs) were calculated to provide a statistical measure of the rate of change in the observed trends over time.

### Statistical Analysis

The GBD 2019 study provide the data encompassing crude estimates, along with their respective 95% uncertainty intervals (UI). The incidence, prevalence, death, and DALY rates were calculated using population data and are reported per 100,000 individuals per year. This analysis delineates the impact of IBD across different demographics, including sex and age groups (segmented into 3-year intervals: 0–9, 10–14, 15–19), as well as by SDI categories. Additionally, exploring the relationship between sociodemographic factors and the burden of IBD among children and adolescents, using the SDI as a reference. All statistical analyses were performed utilizing the open-source R software, version 4.2.1.

## Result

### Overall IBD Burden Among Children and Adolescents

**FIGURE 1:**
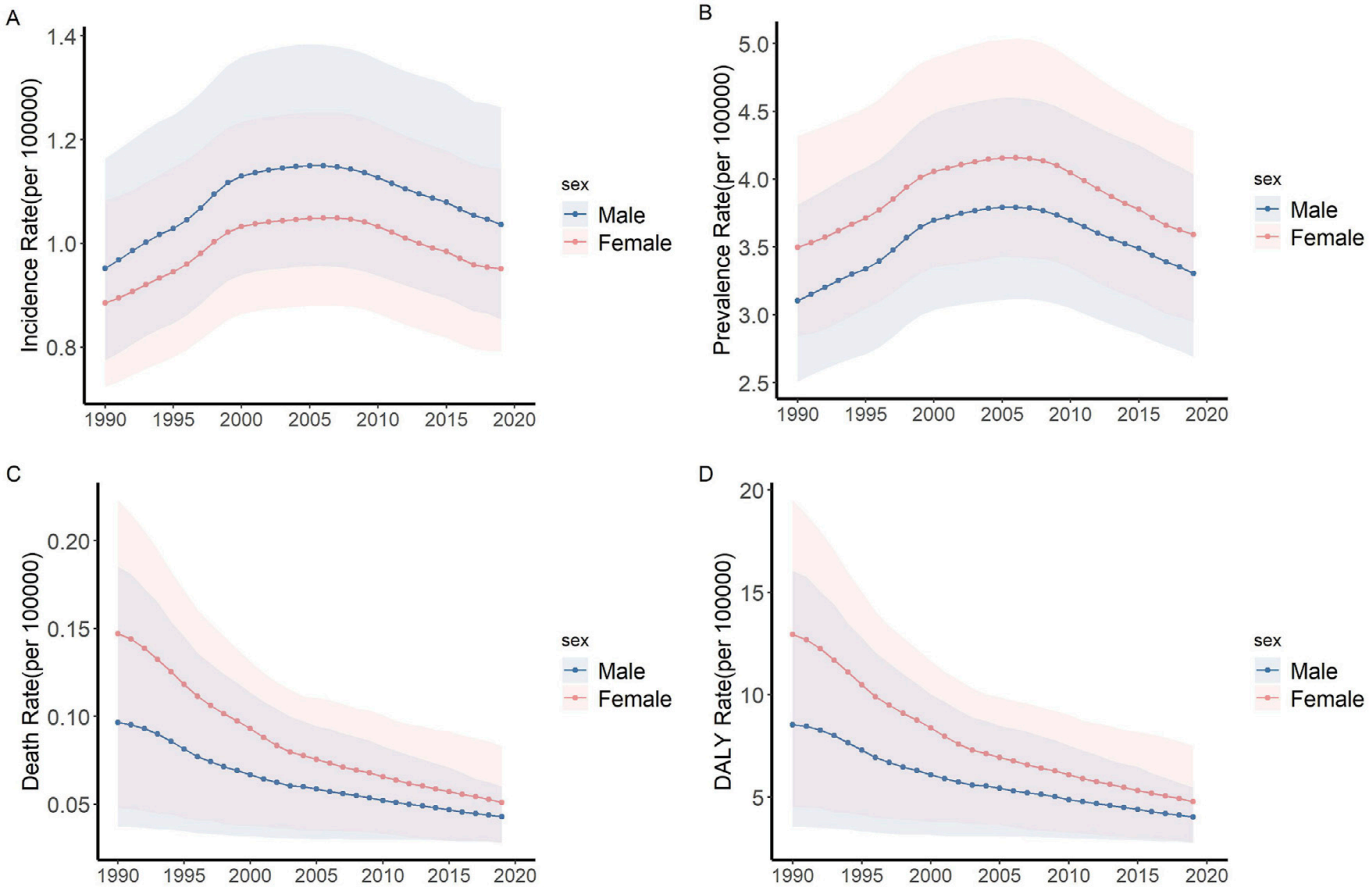
Trends of incidence, prevalence, death and DALY rate of IBD among children and adolescents (Global, 1990–2019). **(A)** Incidence rate. **(B)** Prevalence rate. **(C)** Death rate. **(D)** DALY rate. Abbreviations: IBD, Inflammatory bowel disease; DALY, disability-adjusted life year.

Between 1990 and 2019, the incidence and prevalence of IBD among children and adolescents witnessed a notable increase. The incidence rate marginally escalated from 0.92 (95% UI: 0.75, 1.12) to 0.99 (95% UI: 0.82, 1.20) in this period and the incidence cases escalated from 20,897.42 (95% UI: 17,008.63, 25,520.15) to 25,658.55 (95% UI: 21,268.45, 31,075.58), representing a 22.8% uptick. Concurrently, the prevalence rate escalated from 3.30 (95% UI: 2.67, 4.06) to 3.44 per 100,000 (95% UI: 2.82, 4.20), and the prevalence cases escalated from 74,975.52 (95% UI: 60,678.52, 92,219.20) to 88,828.90 (95% UI: 72,606.82, 108,301.34), representing a 18.5% uptick. Notably, males exhibited consistently higher incidence rate, whereas prevalence rate were higher in females (Figure 1).

During the same period, both mortality and DALY for children and adolescents with IBD exhibited a significant declining trend. Mortality rate decreased from 0.12 (95% UI: 0.05, 0.20) to 0.05 (95% UI: 0.03, 0.06), and the death cases decreased from 2,756.46 (95% UI: 1,162.64, 2,756.46) to 1,208.04 (802.41, 1,651.44), representing a 56.2% downtick. DALY rate dropped from 10.69 (95% UI: 4.76, 17.10) to 4.39 (95% UI: 3.02, 5.83) and the DALY numbers dropped from 243,081.06 (95% UI: 108,233.07, 388,783.07) to 113,119.86 (95% UI: 77,897.11, 150,499.13), representing a 53.5% downtick. Despite females having higher mortality rate initially, their rate of decline (AAPC = −3.6) surpassed that of males (AAPC = −2.8) (Figure 1).

**FIGURE 2:**
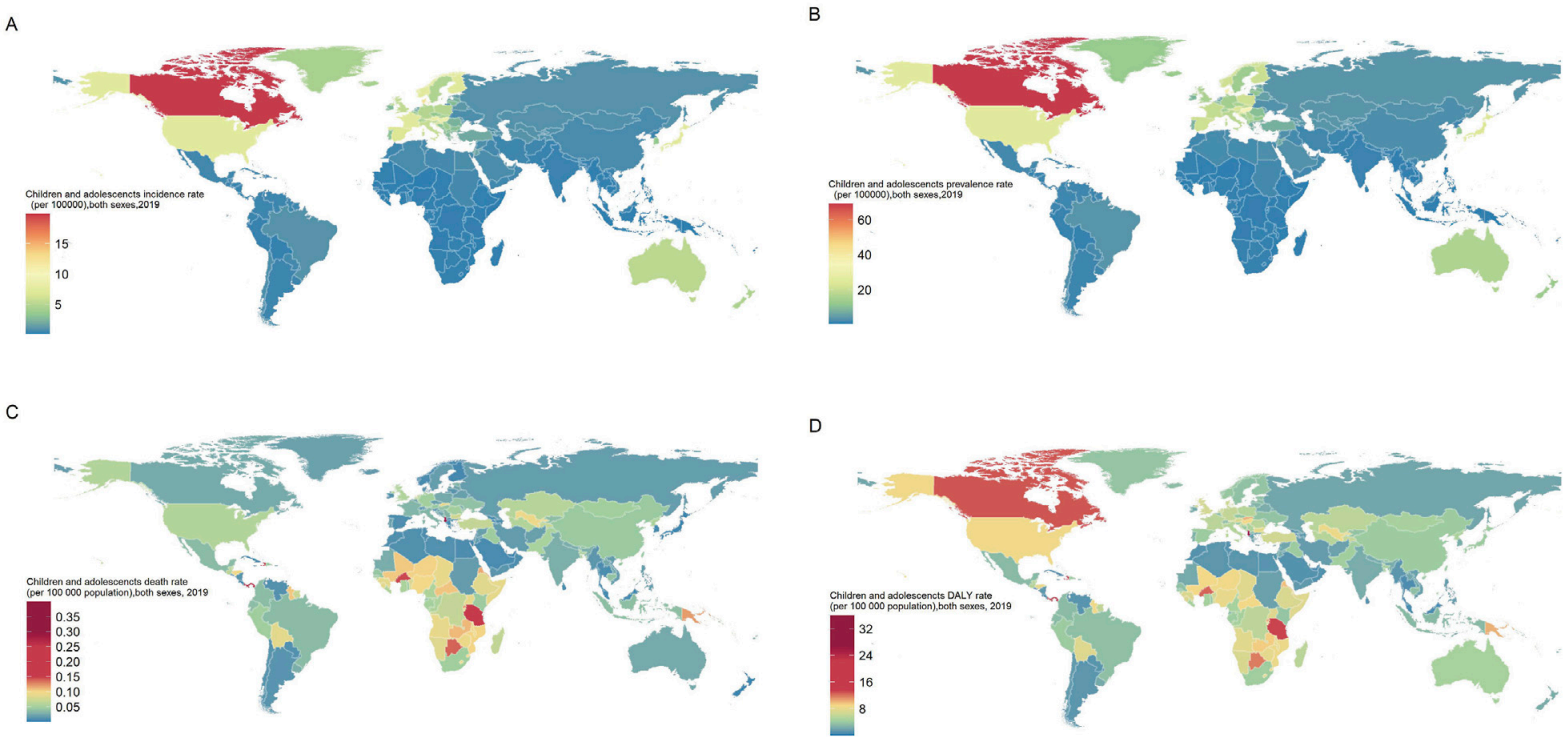
Incidence, prevalence, death and DALY rate of children and adolescents IBD, both sexes, for 204 countries and territories (Global, 2019). **(A)** Incidence rate. **(B)** Prevalence rate. **(C)** Death rate. **(D)** DALY rate. Abbreviations: IBD, Inflammatory bowel disease; DALY, disability-adjusted life year.

In 2019, the burden of IBD varied significantly among children and adolescents across different regions. Nationally, Canada exhibited the highest incidence rate of IBD, registering at 19.88 (95% UI: 18.52, 21.14), while the lowest incidence rate was found in Papua New Guinea, with a value of 0.11 (95% UI: 0.07, 0.15). Additionally, the highest DALY rate was recorded in Albania, amounting to 209.80 (95% UI: 31.91, 300.27), in stark contrast to Cuba, which showed the lowest DALY rate at 0.69 (95% UI: 0.46, 1.16). Upon examining regional data, the Nordic Region exhibited the highest incidence rate, recorded at 7.32 (95% UI: 6.12, 8.76). In contrast, the lowest incidence rate was observed in Sub-Saharan Africa, with a value of 0.17 (95% UI: 0.12, 0.24). However, it is noteworthy that Sub-Saharan Africa reported the highest DALYs, quantified at 9.10 (95% UI: 7.30, 11.33). Conversely, North America demonstrated the lowest DALY rate, at 2.16 (95% UI: 1.65, 2.82) (Figure 2).

### The Association Between IBD Among Children and Adolescents and SDI

**FIGURE 3:**
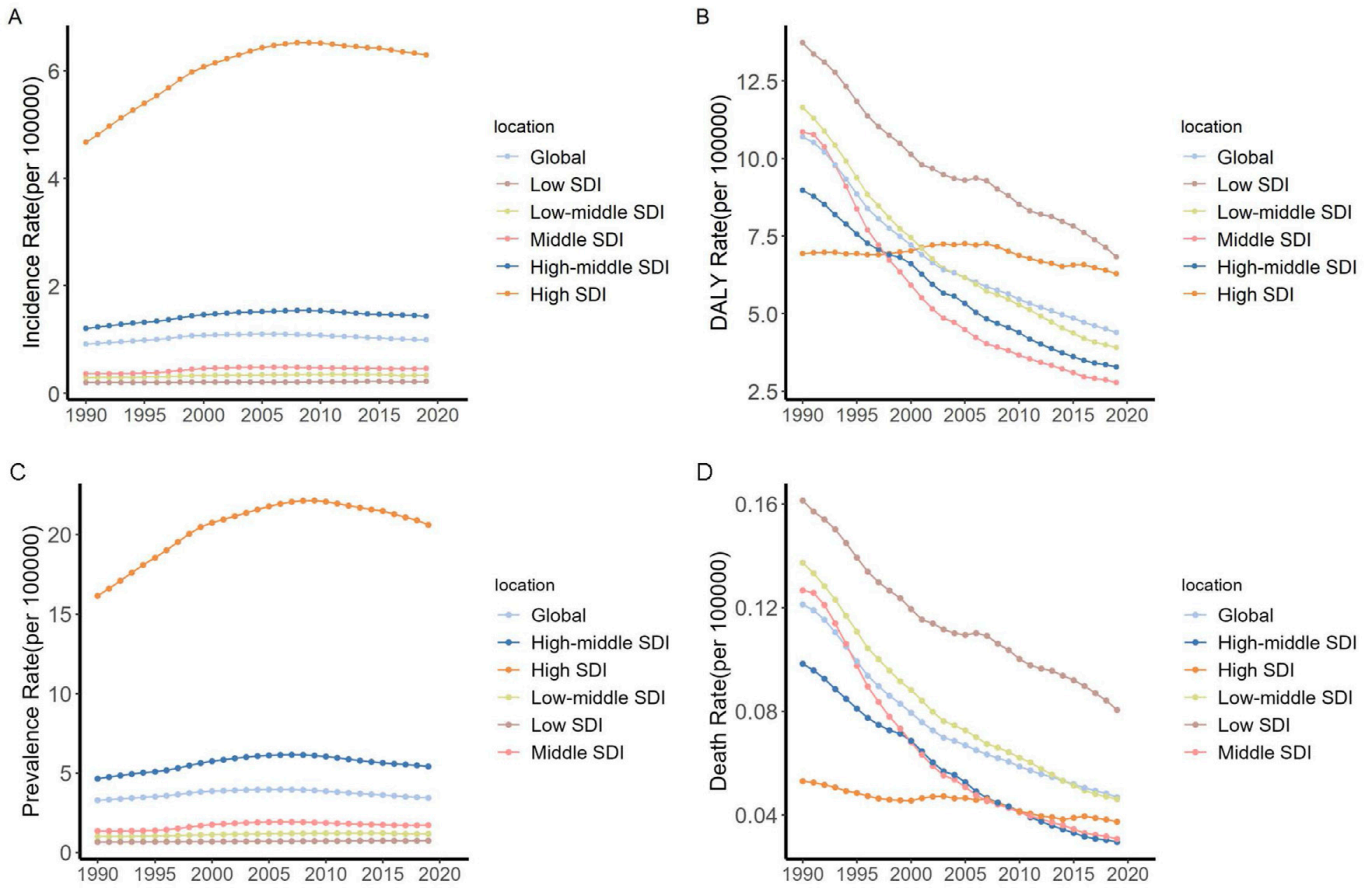
Trends in children and adolescents incidence and DALY rate of IBD by SDI quintile (Global, 1990–2019). **(A)** Incidence rate. **(B)** DALY rate. **(C)** Prevalence rate. **(D)** Death rate. Abbreviations: IBD, Inflammatory bowel disease; DALY, disability-adjusted life year; SDI, Sociodemographic Index.

Regions with a high SDI exhibited significantly higher incidence rate of pediatric and adolescent IBD compared to the rest of the world. From 1990 to 2019, the incidence increased most rapidly in high SDI, increased from 4.67 per 100,000 (95% UI: 3.93–5.54) to 6.30 per 100,000 (95% UI: 5.42–7.32), marking a 34.83% rise. Global DALY rate has been declining; however, this change has been the slowest in high SDI regions (AAPC = −0.09). As of 2019, both high and low SDI regions reported relatively high levels of DALY (Figure 3).

**FIGURE 4:**
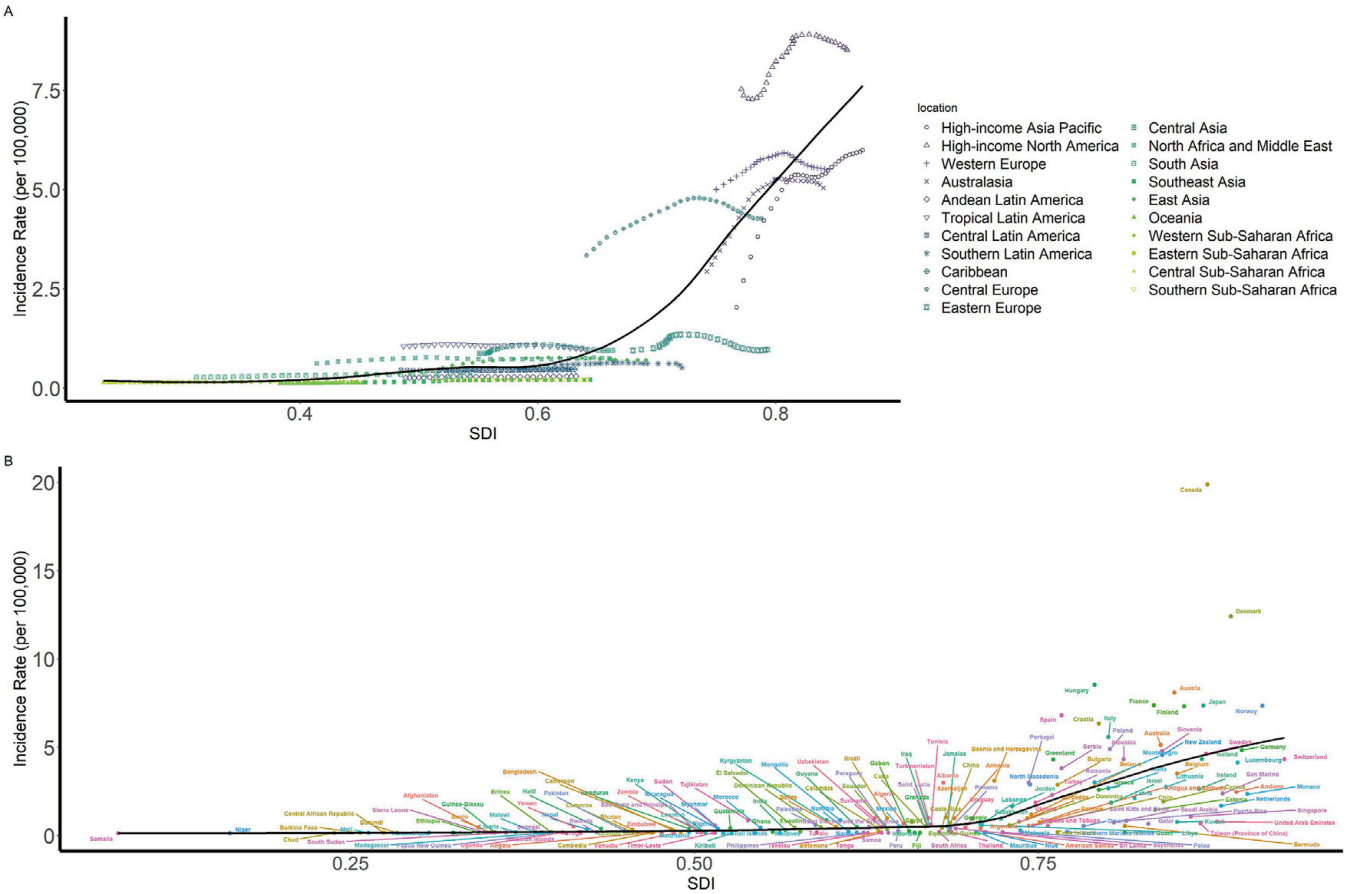
Children and adolescents incidence rate of IBD by SDI (Global, 1990–2019). **(A)** Incidence rate globally and for 21 GBD regions by SDI, 1990–2019 **(B)** Incidence rate for 204 countries and territories by SDI, 2019. Abbreviations: IBD, Inflammatory bowel disease; DALY, disability-adjusted life year; SDI, Sociodemographic Index.

We further explored the association between the incidence rate of diseases in children and adolescents and the SDI. Figure 4 demonstrates a correlation between SDI and incidence rate. It is worth noting that at the regional level, the incidence rate in high-income areas of North America consistently exceeded the expected levels based on SDI from 1990 to 2019. Within individual countries, in 2019, the incidence rate in certain countries were significantly higher than the expected SDI for that year, such as Canada and Denmark.

### Disease Burden of Among Early-Onset IBD and Its Time Trend

**FIGURE 5:**
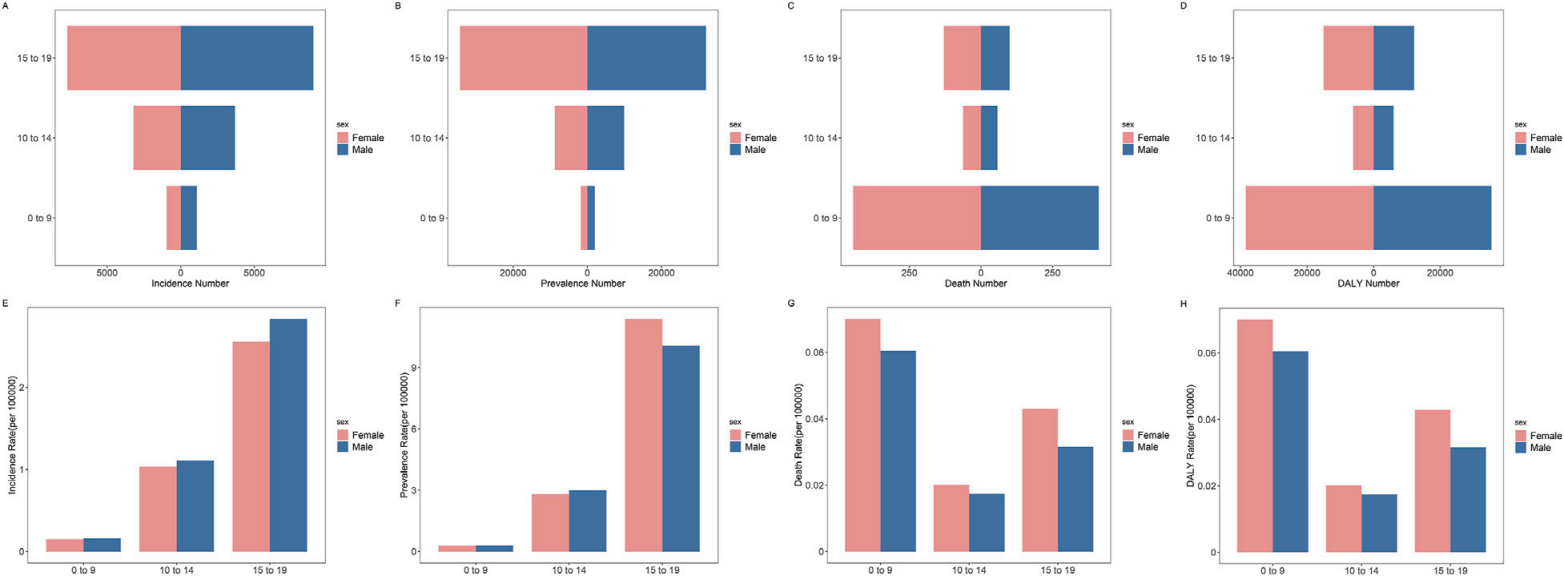
Age distribution of global children and adolescents incidence cases, incidence rate, prevalence cases, prevalence rate, death cases, death rate, DALY number and DALY rate of IBD by sex (Global, 2019). **(A)** Incidence cases. **(B)** Prevalence cases. **(C)** Death cases. **(D)** DALY number. **(E)** Incidence rate. **(F)** Prevalence rate. **(G)** Death rate. **(H)** DALY rate. Abbreviations: IBD, Inflammatory bowel disease; DALY, disability-adjusted life year.

Our analysis further delved into the burden of early-onset IBD. In 2019, within the age bracket of 0–9 years, there were recorded incidence cases amounting to 2,053.52 (95% UI: 1,575.62, 2,677.49), representing 8.0% of the overall IBD cases among children and adolescents. Notably, the DALY attributed to early-onset IBD reached 73,797.46 (95% UI: 43,655.86, 105,998.63), marking the highest burden of disease within the IBD-affected pediatric and adolescent population (Figure 5).

**FIGURE 6:**
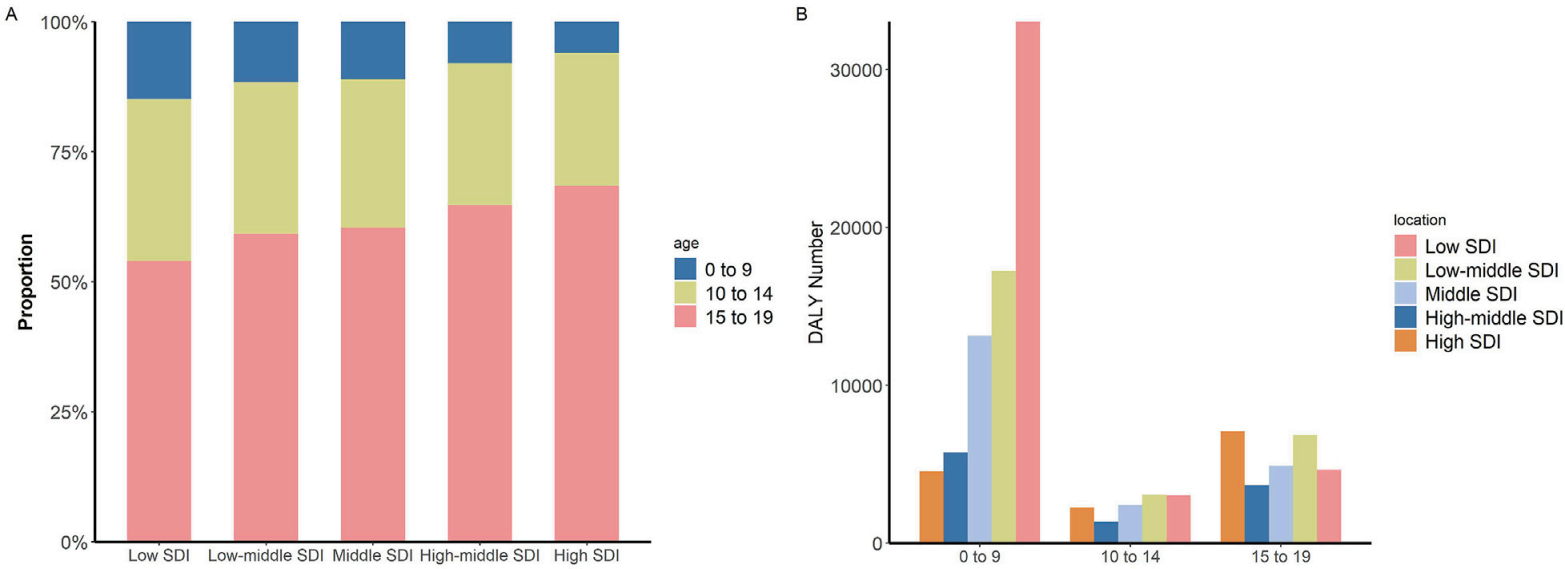
Children and adolescents incidence and DALY numbers of IBD (Global, 2019). **(A)** Proportion of incidence cases in different SDI regions by age groups. **(B)** DALY numbers in different age groups by SDI regions. Abbreviations: IBD, Inflammatory bowel disease; DALY, disability-adjusted life year; SDI, Sociodemographic Index.

In 2019, across various SDI regions, the lowest SDI areas exhibited the highest proportion of incidence cases of IBD in the 0–9 age group among children and adolescents, accounting for 14.9%. Furthermore, as the SDI increased, there was a gradual decline in the proportion of the 0–9 age group within the overall incidence cases of IBD among children and adolescents. In the 0–9 age group, compared with other SDI regions, the DALY numbers in low SDI regions were the highest 33,033.27 (95% UI: 11,373.40, 58,627.61), accounting for 44.8% of the total DALY numbers for this age group (Figure 6).

The joinpoint regression analysis, illustrating the incidence rate trends of IBD among children and adolescents between 1990 and 2019, is presented in Supplementary Figure S7. Throughout this period, the incidence rate of IBD within this demographic demonstrated a consistent upward trend, with an Average Annual Percent Change (AAPC) of +0.3 (95% CI: 0.2, 0.3). Specifically, early-onset IBD experienced a modest increase, reflected by an AAPC of +0.1 (95% CI: 0.1, 0.2). Conversely, an examination of DALY rate revealed a declining trend in IBD among children and adolescents, characterized by an AAPC of −3.0 (95% CI: −2.9, −3.0), and the 0–9 age group displaying a notable decrease in DALY rate, characterized by an AAPC of −3.6 (95% CI: −3.8, −3.5). Prevalence and mortality rate was detailed in the Supplementary Material (Supplementary Figures S7–S29).

## Discussion

We conducted a comprehensive and detailed analysis of the incidence, prevalence, death, and DALY of IBD among children and adolescents aged 0–19 globally. Our study reveals the burden of IBD among children and adolescents on a global scale, highlighting significant variations in these indicators across different regions and populations. Our results indicate a downward trend in the global burden of disease when countries are classified by SDI. Over the past 30 years, significant progress has been made in treating IBD in children and adolescents worldwide. However, the decline in the burden of disease has been slowest in high SDI regions, suggesting that the measures implemented in these regions may be insufficient, further measures are needed to reduce the burden of IBD in children and adolescents.

A large number of studies have indicated a continuous increase in the incidence and prevalence of adult IBD across various regions [23, 24]. However, epidemiological research on pediatric IBD worldwide has been limited [12]. Although the increase in the incidence and prevalence of IBD among children and adolescents was slight during the study period, the increase in the incidence cases and prevalence cases of IBD in children and adolescents was more than 20% due to the global population growth. In countries and regions level, were found that Canada has the highest incidence rate of pediatric IBD, following closely, Denmark stands as the country with the second-highest incidence rate. In contrast, the incidence rate in Asia, Southern Latin America and Africa were significantly lower. A systematic review reported a similar finding that the highest incidence rate of pediatric IBD were observed in Canada, Northern Europe, and New Zealand, while the lowest were in Southern Europe, Africa, Asia, and South America [25]. Furthermore, our study reveals a positive correlation between the incidence of IBD among children and adolescents and the SDI. Specifically, regions with a high SDI exhibit an incidence rate of 19.39, indicating that higher socio-demographic development is associated with an increased incidence of IBD in this population group [26–28].

Our study found a significant decline in the mortality and DALY associated with IBD among children and adolescents in all SDI regions expect for high SDI region from 1990 to 2019. This decline could likely be attributed to the relatively stable incidence and the use of emerging medications such as immunosuppressants and biologics [23, 27]. Despite high SDI regions having advanced medical technology, the increase in incidence and the rising number of cases have prevented a significant decline in overall DALYs. Compared to IBD in adulthood, the onset of the disease in childhood may present with higher disease activity and a more complex course [12]. When treating children and adolescents with IBD, it is essential to consider the impact of the disease on growth, skeletal health, and psychosocial functioning [29–31]. Although low SDI areas show a clear downward trend in DALYs, they still have the highest overall DALY burden. Our research indicates that in regions with a low SDI, such as sub-Saharan Africa, despite having the lowest incidence rate, they exhibit the highest global DALY, suggesting a significant underemphasis on IBD in children and adolescents in less developed countries, leading to a considerable disease burden. There is currently a lack of clinical evidence related to IBD in children and adolescents, making the treatment and management of these patients more challenging, especially in the low SDI region [12, 29]. Compared to low SDI areas, although the absolute number of DALYs in high SDI areas was lower, the trend in DALYs remained relatively flat during the study period, indicating no significant improvement in disease burden. Countries with high SDI are in the third of four epidemiological phases in the global evolution of IBD, the disease worsening phase. Since IBD is a chronic disease with no cure, as long as the incidence exceeds the mortality rate, the prevalence will steadily increase. Despite the higher level of medical care in high SDI areas, the disease burden remains high due to the increasing number of patients [32]. The imperative to intensify research and clinical attention towards IBD in pediatric and adolescent populations is underscored by the considerable disease burden this group endures. Enhancing the focus on these younger cohorts is essential for both mitigating the immediate impacts of the disease and reducing long-term complications, especially in regions with a higher disease burden.

According to the international diagnostic standards such as the Montreal and Paris classifications, IBD diagnosed before the age of 10 is referred to as early-onset IBD [16, 31]. Early-onset IBD is characterized by early onset, severe disease course, difficult-to-control diarrhea, significant impact on growth and development, frequent severe perianal lesions, and often hidden genetic defects, with earlier onset predicting worse prognosis [33]. Studies have indicated that patients with early-onset IBD may differ in etiology and disease manifestation from non-early-onset pediatric IBD patients [31]. Therefore, in our research, we compared the groups aged 0–9 with those aged 10–14 and 15–19 years. Compared to non-early-onset IBDs, early-onset IBD has a lower incidence rate but higher mortality and DALY rate. Furthermore, compared to other SDI regions, early-onset IBD in low SDI regions has higher mortality and DALY rate. Due to the inflammatory mechanisms of early-onset IBD caused by gene mutations and non-gene mutations are different, conventional immunosuppressants and medications like mesalamine have minimal effect on early-onset IBD caused by genetic mutations [17, 34]. Joinpoint analysis results indicate that the reduction in mortality and DALY rate for early-onset IBD far exceeds that of non-early-onset pediatric IBD, with an AAPC reaching −3.6%. Studies have shown that early-onset IBD patients diagnosed at the initial stages of the disease have relatively better treatment outcomes and require significantly less subsequent healthcare and surgical interventions compared to those diagnosed after the age of 10 [12]. There is still a need to enhance the focus on early-onset IBD, to improve the diagnostic capabilities and enhance the quality of life for patients with early-onset IBD.

This study provides a thorough evaluation of the burden of pediatric IBD from 1990 to 2019, including incidence, prevalence, mortality, and DALY, with an in-depth analysis of early-onset IBD. Pediatric IBD represents a distinct group, and based on our findings, there are significant differences compared to adults, necessitating increased attention in epidemiological, basic, and clinical research. Our work has filled the gap in understanding the disease burden of IBD. Nevertheless, our research has several limitations. First, GBD data do not distinguish between CD and UC, requiring further epidemiological analysis of each subtype [23, 27]. Second, our study is constrained by the inherent limitations related to the GBD study, including the quality and accessibility of the original data. Due to the limited research on pediatric and adolescent IBD, GBD data may exhibit certain biases [22]. Due to the feasibility of data collection, GBD data reflects not only the burden of disease but also the level of local medical care and development. In some underdeveloped areas, data collection can be challenging, particularly for rare diseases such as IBD, which require a high level of medical expertise for diagnosis. In data-scarce locations, GBD had to rely on predictive covariates and spatiotemporal trends. For non-fatal models, where data were especially scarce, estimates for some regions were determined entirely by global trends and associations with income and healthcare access, along with data from a single country. This is reflected in wider UIs in these locations, suggesting that extra caution should be applied when interpreting estimates for these areas. Additionally, with each iteration, the GBD database becomes increasingly refined, enhancing its alignment with real-world conditions. This iterative process continuously improves data quality, making the estimates more reliable over time [27]. Finally, current research categorizes pediatric IBD into neonatal IBD, infantile IBD, very early-onset IBD, and early-onset IBD. However, due to the constraints of GBD data, our focus was solely on early-onset IBD.

### Conclusion

As the global burden of IBD among children and adolescents continues to escalate, enhancing the diagnosis, treatment, and management of children and adolescents IBD becomes imperative. Over the past 30 years, significant progress has been made globally in treating IBD in children and adolescents. However, in high SDI areas, the decrease in disease burden has not been significant, suggesting that current measures may be insufficient and that further action is needed to reduce the burden of IBD among children and adolescents in these populations.

## Data Availability

Data can be obtained from the Global Health Data Exchange Global Burden of Disease Results Tool (https://ghdx.healthdata.org/gbd-results-tool).

## Abbreviations

GBD, Global Burden of Disease; IBD, Inflammatory bowel disease; DALY, Disability-Adjusted Life Years; APC, annual percentage change; AAPC, average annual percent change.
