# Supplementary material for: Global Burden of Inflammatory Bowel Disease Among Children and Adolescents: A Comprehensive Analysis (1990–2019)
Source: Int J Public Health. 2024 Sep 9;69:1607440. doi: 10.3389/ijph.2024.1607440 (PMC11417169; doi:10.3389/ijph.2024.1607440)
Supplement: Supplementary file 5 [file DataSheet1.DOCX]

**Supplemental Materials**

**Supplemental method**

The Global Burden of Disease (GBD) study provides comprehensive estimates of incidence, prevalence, mortality, years of life lost (YLLs), years lived with disability (YLDs), and disability-adjusted life-years (DALYs) for 369 diseases and injuries across both sexes and in 204 countries and territories. The input data are sourced from various platforms, including censuses, household surveys, civil registration and vital statistics, disease registries, health service usage, air pollution monitors, satellite imagery, disease notifications, and additional sources.

Cause-specific death rates and fractions were determined using the Cause of Death Ensemble model and spatiotemporal Gaussian process regression. These rates were adjusted to align with total all-cause deaths calculated within the GBD framework, which includes population, fertility, and mortality estimates. YLLs were derived by multiplying deaths by the standard life expectancy at each age. A Bayesian meta-regression modelling tool, DisMod-MR 2.1, ensured consistency among incidence, prevalence, remission, excess mortality, and cause-specific mortality for most conditions. YLDs were calculated by multiplying prevalence estimates by disability weights assigned to the distinct sequelae of diseases and injuries. GBD results were contextualized using the Socio-demographic Index (SDI), which combines income per capita, years of schooling, and fertility rates in females under 25 years. Uncertainty intervals (UIs) were computed for each metric based on the 25th and 975th ordered values from 1,000 draws of the posterior distribution.

Crohn’s disease and ulcerative colitis, the two main types of inflammatory bowel disease (IBD) studied, are diagnosed through endoscopy, imaging studies, or biopsy in patients exhibiting relevant clinical symptoms. In certain cases where a definitive diagnosis cannot distinguish between Crohn’s disease and ulcerative colitis, a diagnosis of indeterminate colitis is used, potentially remaining in place until more specific diagnostic features emerge. According to the International Classification of Diseases, Tenth Revision (ICD-10), the codes are K50 for Crohn’s disease, K51 for ulcerative colitis, and K52 for indeterminate colitis.

**Data processing**

A crucial step in the GBD analytical process is the correction for known biases. This involves redistributing deaths classified under unspecified codes to more specific disease categories and adjusting data collected using alternative case definitions or measurement methods to align with the reference method.

**Cause of death redistribution**

Vital registration with medical certification of cause of death is a crucial resource for the GBD cause of death analysis in many countries. Cause of death data, obtained using various revisions of the International Classification of Diseases and Injuries (ICD), were mapped to the GBD cause list. However, many deaths are recorded under causes that cannot serve as the underlying cause of death (e.g., cardiopulmonary failure) or are inadequately specified (e.g., injury from undetermined intent). As part of the GBD data processing, these deaths were reassigned to the most probable underlying causes.

Redistribution algorithms fall into three categories: proportionate redistribution, fixed proportion redistribution based on published studies or expert judgment, and statistical algorithms. For GBD 2019, data from 116 million deaths attributed to multiple causes were analyzed to develop more empirical redistribution algorithms. These algorithms addressed conditions such as sepsis, heart failure, pulmonary embolism, acute kidney injury, hepatic failure, acute respiratory failure, pneumonitis, and five intermediate causes in the central nervous system (hydrocephalus, toxic encephalopathy, compression of brain, encephalopathy, and cerebral edema).

For unspecified injuries, GBD employed a method similar to that used for intermediate cause redistribution. This method analyzed the pattern of injury codes in the causal chain, with ICD codes X59 (“exposure to unspecified factor”) and Y34 (“unspecified event, undetermined intent”) used as indicators, along with GBD injury causes, to identify the underlying cause of death. These new algorithms significantly altered the causes to which these intermediate outcomes were redistributed, leading to important updates in the classification of causes of death.

**Mortality estimates**

To model mortality due to inflammatory bowel disease (IBD), the GBD study utilized the causes of death database, which compiles data from vital registration and verbal autopsy records. The methodology for processing these causes of death data has been documented previously. Data were identified as outliers if the redistribution of garbage codes and noise reduction, combined with small sample sizes, resulted in implausible cause fractions, or if the data conflicted with well-established temporal or age trends.

We modeled IBD-related deaths using the Cause of Death Ensemble model (CODEm), incorporating inputs from the causes of death database and location-specific covariates. Separate global and data-rich models for each sex were hybridized to produce unadjusted results. These results were then refined and adjusted to align with all-cause mortality levels for each age-sex-year location using the cause of death correction procedure (CODCorrect), ultimately deriving the final years of life lost (YLLs) due to IBD. The method for propagating uncertainty followed the approach used in previous GBD publications. Each step in the computation process was represented by a distribution stored in 1,000 draws, which informed every subsequent step. Final estimates were calculated as the mean of these 1,000 draws, with the 95% uncertainty intervals (UIs) based on the 25th and 975th ranked values.

The percentage change between any two years of GBD estimates was calculated at the draw level. For instance, each of the 1,000 draws for 2017 was compared with the corresponding draw for 1990, producing 1,000 percent change draws. The mean of these draws, along with the 25th and 975th ordered draws, were used to report the percentage change as the mean, lower UI limit, and upper UI limit, respectively.

**Non-fatal estimates**

To estimate the non-fatal burden of IBD, the GBD study used two separate databases: one for Crohn’s disease and another for ulcerative colitis. These databases included data from literature, hospital discharges, and claims data. Claims data were used to link multiple inpatient and outpatient claims to a single individual. Prevalent cases were identified if an individual had at least one inpatient or outpatient encounter with a relevant ICD code in any diagnosis field.

Data from hospital discharges were adjusted using correction factors derived from claims data. This adjustment process converted encounters into estimates of cases, accounted for some facilities only providing primary diagnostic codes, and estimated outpatient cases based on inpatient data. In brief, a systematic review of the literature was conducted to identify studies on the prevalence and incidence of IBD. Studies were excluded if they were not representative of the national population or if they employed insufficient or inappropriate sampling methods. Reviews were also excluded from the search results to ensure that only primary data sources were considered.

**Correcting for non-reference case definitions or measurement methods**

In previous cycles of the GBD study, data reported using alternative case definitions or measurement methods were corrected to align with the reference definition or measurement method. This adjustment was primarily implemented within Bayesian meta-regression models. For instance, in DisMod-MR, population data were simultaneously modeled both as a function of country-specific covariates reflecting true variation in rates and as a function of indicator variables that accounted for alternative measurement methods.

To enhance transparency and standardize the methods in GBD 2019, correction factors for alternative case definitions or measurement methods were estimated using network meta-regression. This approach included only data where two methods were assessed either in the same location and time period or in the exact same population. The data also encompassed validation studies where two methods were compared in populations that were not necessarily representative samples of the general population. This approach aimed to ensure that the estimates were as accurate and comparable as possible across different methodologies.

**Clinical informatics**

Clinical informatics data encompass inpatient admissions, outpatient visits (including general practitioner visits), and health insurance claims. Several data processing steps were undertaken to ensure comprehensive data coverage. Inpatient hospital data with a single diagnosis were adjusted to account for non-primary diagnoses and associated outpatient care.

For each GBD cause that utilized clinical data, ratios of non-primary to primary diagnosis rates were extracted from claims data in the USA, Taiwan (province of China), New Zealand, and the Philippines, as well as from the USA Healthcare Cost and Utilization Project inpatient data. Additionally, ratios of outpatient to inpatient care for each cause were extracted from claims data in the USA and Taiwan (province of China). These ratios were logarithmically modeled by age and sex using MR-BRT (Meta-Regression-Bayesian Regularised Trimmed), a Bayesian meta-regression tool.

To address incomplete healthcare access in populations where not every individual with a disease or injury is captured in administrative clinical records, we adjusted the admission rates using a scalar derived from the Healthcare Access and Quality Index. This transformation aimed to account for variations in healthcare access and ensure that the data more accurately reflected the true burden of disease.

**Modelling**

For most diseases and injuries, processed data are modeled using standardized tools to generate estimates for each quantity of interest by age, sex, location, and year. The three main standardized tools are the Cause of Death Ensemble model (CODEm), spatiotemporal Gaussian process regression (ST-GPR), and DisMod-MR.

Briefly, CODEm is a highly systematic tool that analyzes cause of death data by employing an ensemble of different modeling methods for rates or cause fractions, using various covariates that perform best in out-of-sample predictive validity tests. DisMod-MR is a Bayesian meta-regression tool that evaluates all available data on disease incidence, prevalence, remission, and mortality, ensuring consistency among these epidemiological parameters. ST-GPR consists of regression methods that leverage information across locations and over time for single metrics of interest, such as risk factor exposure or mortality rates, enhancing the robustness and reliability of the estimates.

**Socio-demographic Index, annual rate of change, and data presentation**

The Socio-demographic Index (SDI) is a composite indicator developed by GBD researchers to reflect a country's development status and its correlation with health outcomes. It combines lag-distributed income per capita, average years of schooling, and the fertility rate in females under 25 years. The SDI is the geometric mean of three indices ranging from 0 to 1: total fertility rate under the age of 25 (TFU25), mean education for those aged 15 and older (EDU15+), and lag-distributed income (LDI) per capita. An SDI value of 0 represents a theoretical minimum level of development related to health, while a value of 1 signifies a theoretical maximum level.

For changes over time, GBD presents annualized rates of change as the difference in the natural logarithm of the values at the start and end of the time interval, divided by the number of years in the interval. The GBD study examines the relationship between SDI and the annualized rate of change in age-standardized DALY rates for all causes except HIV/AIDS, natural disasters, and war and conflict, by country or territory for the periods 1990–2010 and 2010–2019. DALYs due to HIV/AIDS were excluded from the analysis to prevent their significant impact in some regions from obscuring trends in other causes. Similarly, DALY rates from natural disasters and war and conflict were subtracted to avoid skewing the disease burden trends due to these sudden and dramatic events.

As a measure of the epidemiological transition, GBD presents the ratio of years lived with disability (YLDs) due to non-communicable diseases and injuries to the total burden in DALYs. For each metric, GBD provides 95% uncertainty intervals (UIs), based on the 25th and 975th ordered values from 1,000 draws of the posterior distribution.

**Supplemental Figure**

Figure S1 Incidence rate of IBD among children and adolescents globally and for 21 GBD regions by SDI, 1990–2019.

Figure S2 Incidence rate of IBD among children and adolescents for 204 countries and territories by SDI, 2019.

Figure S3 DALY rate of IBD among children and adolescents globally and for 21 GBD regions by SDI, 1990–2019.

Figure S4 DALY rate of IBD among children and adolescents for 204 countries and territories by SDI, 2019.

Figure S5 Children and adolescents prevalence cases of IBD in different age groups by SDI regions, 2019.

Figure S6 Children and adolescents death cases of IBD in different age groups by SDI regions, 2019.

Figure S7 Joinpoint regression analysis of the early-onset IBD and non-early-onset IBD incidence and DALY rate globally from 1990 to 2019. (A) incidence rate. (B) DALY rate.

Figure S8 Joinpoint regression analysis of the early-onset IBD and non-early-onset IBD incidence rate for low SDI regions from 1990 to 2019.

Figure S9 Joinpoint regression analysis of the early-onset IBD and non-early-onset IBD incidence rate for low-middle SDI regions from 1990 to 2019.

Figure S10 Joinpoint regression analysis of the early-onset IBD and non-early-onset IBD incidence rate for middle SDI regions from 1990 to 2019.

Figure S11 Joinpoint regression analysis of the early-onset IBD and non-early-onset IBD incidence rate for high-middle SDI regions from 1990 to 2019.

Figure S12 Joinpoint regression analysis of the early-onset IBD and non-early-onset IBD incidence rate for high SDI regions from 1990 to 2019.

Figure S13 Joinpoint regression analysis of the early-onset IBD and non-early-onset IBD prevalence rate for low SDI regions from 1990 to 2019.

Figure S14 Joinpoint regression analysis of the early-onset IBD and non-early-onset IBD prevalence rate for low SDI regions from 1990 to 2019.

Figure S15 Joinpoint regression analysis of the early-onset IBD and non-early-onset IBD prevalence rate for low SDI regions from 1990 to 2019.

Figure S16 Joinpoint regression analysis of the early-onset IBD and non-early-onset IBD prevalence rate for low SDI regions from 1990 to 2019.

Figure S17 Joinpoint regression analysis of the early-onset IBD and non-early-onset IBD prevalence rate for low SDI regions from 1990 to 2019.

Figure S18 Joinpoint regression analysis of the early-onset IBD and non-early-onset IBD prevalence for low SDI regions from 1990 to 2019.

Figure S19 Joinpoint regression analysis of the early-onset IBD and non-early-onset IBD death rate for low SDI regions from 1990 to 2019.

Figure S20 Joinpoint regression analysis of the early-onset IBD and non-early-onset IBD death rate for low SDI regions from 1990 to 2019.

Figure S21 Joinpoint regression analysis of the early-onset IBD and non-early-onset IBD death rate for low SDI regions from 1990 to 2019.

Figure S22 Joinpoint regression analysis of the early-onset IBD and non-early-onset IBD death rate for low SDI regions from 1990 to 2019.

Figure S23 Joinpoint regression analysis of the early-onset IBD and non-early-onset IBD death rate for low SDI regions from 1990 to 2019.

Figure S24 Joinpoint regression analysis of the early-onset IBD and non-early-onset IBD death rate for low SDI regions from 1990 to 2019.

Figure S25 Joinpoint regression analysis of the early-onset IBD and non-early-onset IBD DALY rate for low SDI regions from 1990 to 2019.

Figure S26 Joinpoint regression analysis of the early-onset IBD and non-early-onset IBD DALY rate for low SDI regions from 1990 to 2019.

Figure S27 Joinpoint regression analysis of the early-onset IBD and non-early-onset IBD DALY rate for low SDI regions from 1990 to 2019.

Figure S28 Joinpoint regression analysis of the early-onset IBD and non-early-onset IBD DALY rate for low SDI regions from 1990 to 2019.

Figure S29 Joinpoint regression analysis of the early-onset IBD and non-early-onset IBD DALY rate for low SDI regions from 1990 to 2019.


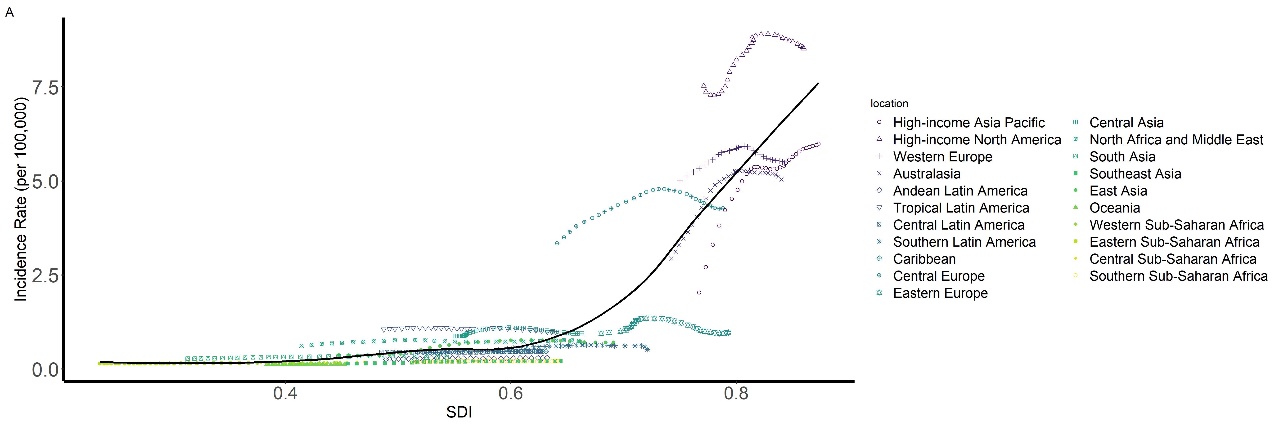


Figure S1 Incidence rate of IBD among children and adolescents globally and for 21 GBD regions by SDI, 1990–2019.


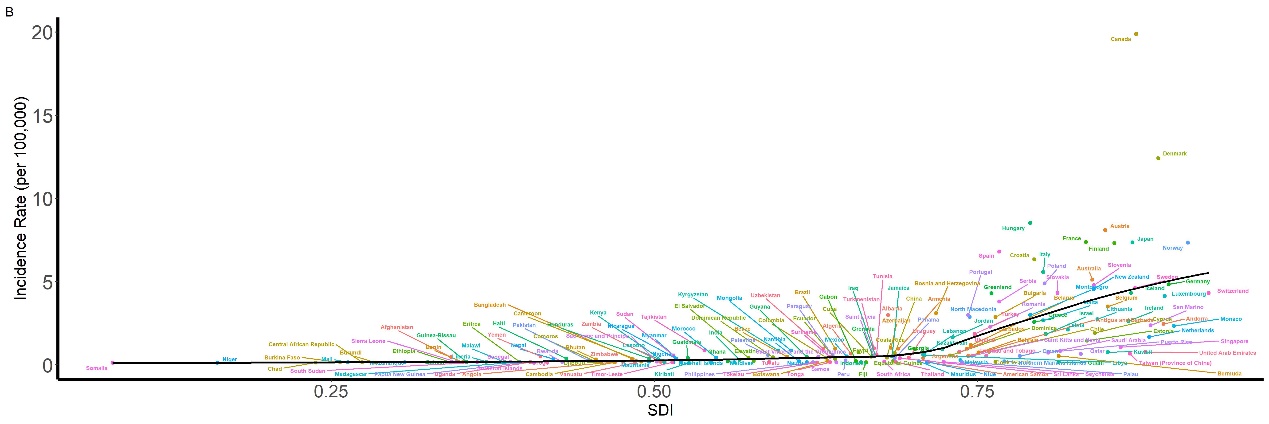


Figure S2 Incidence rate of IBD among children and adolescents for 204 countries and territories by SDI, 2019.


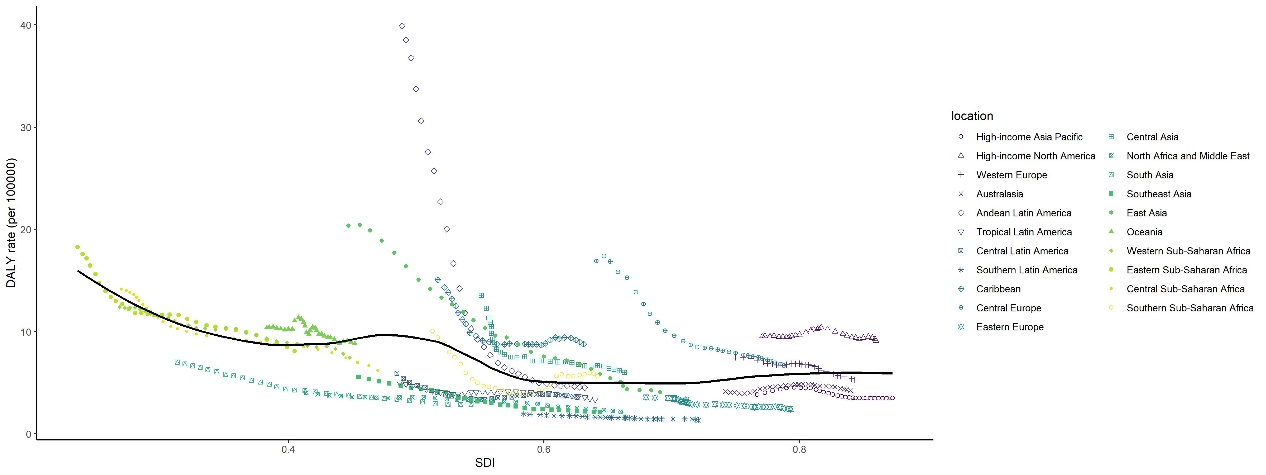


Figure S3 DALY rate of IBD among children and adolescents globally and for 21 GBD regions by SDI, 1990–2019.


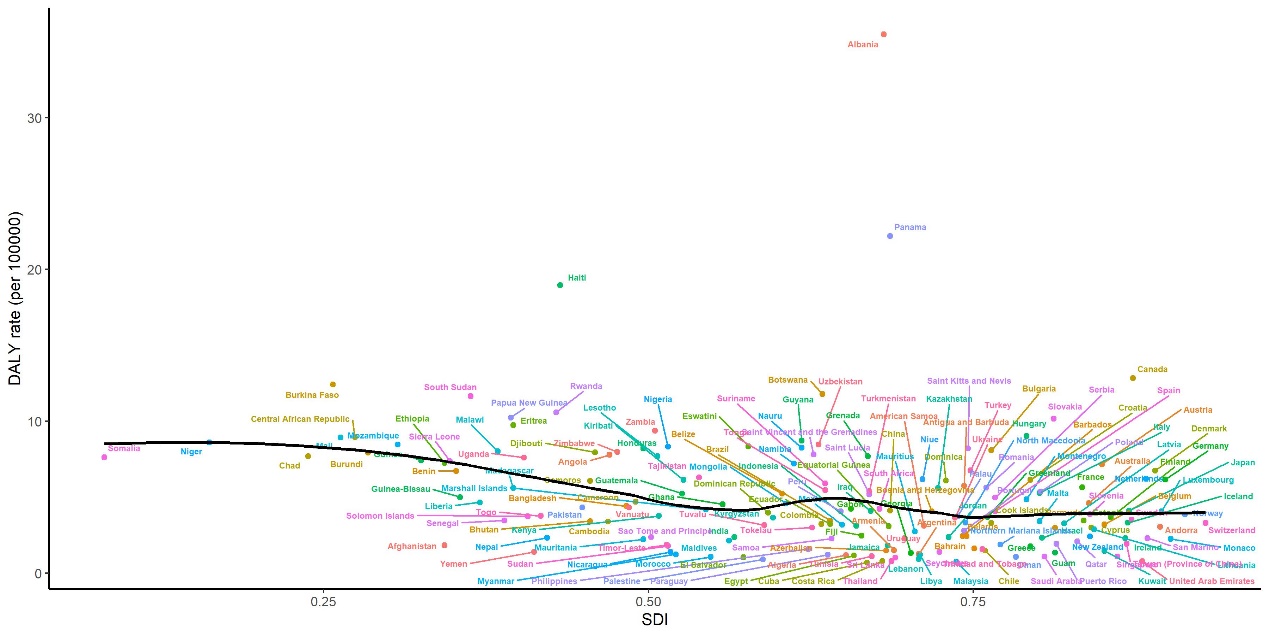


Figure S4 DALY rate of IBD among children and adolescents for 204 countries and territories by SDI, 2019.


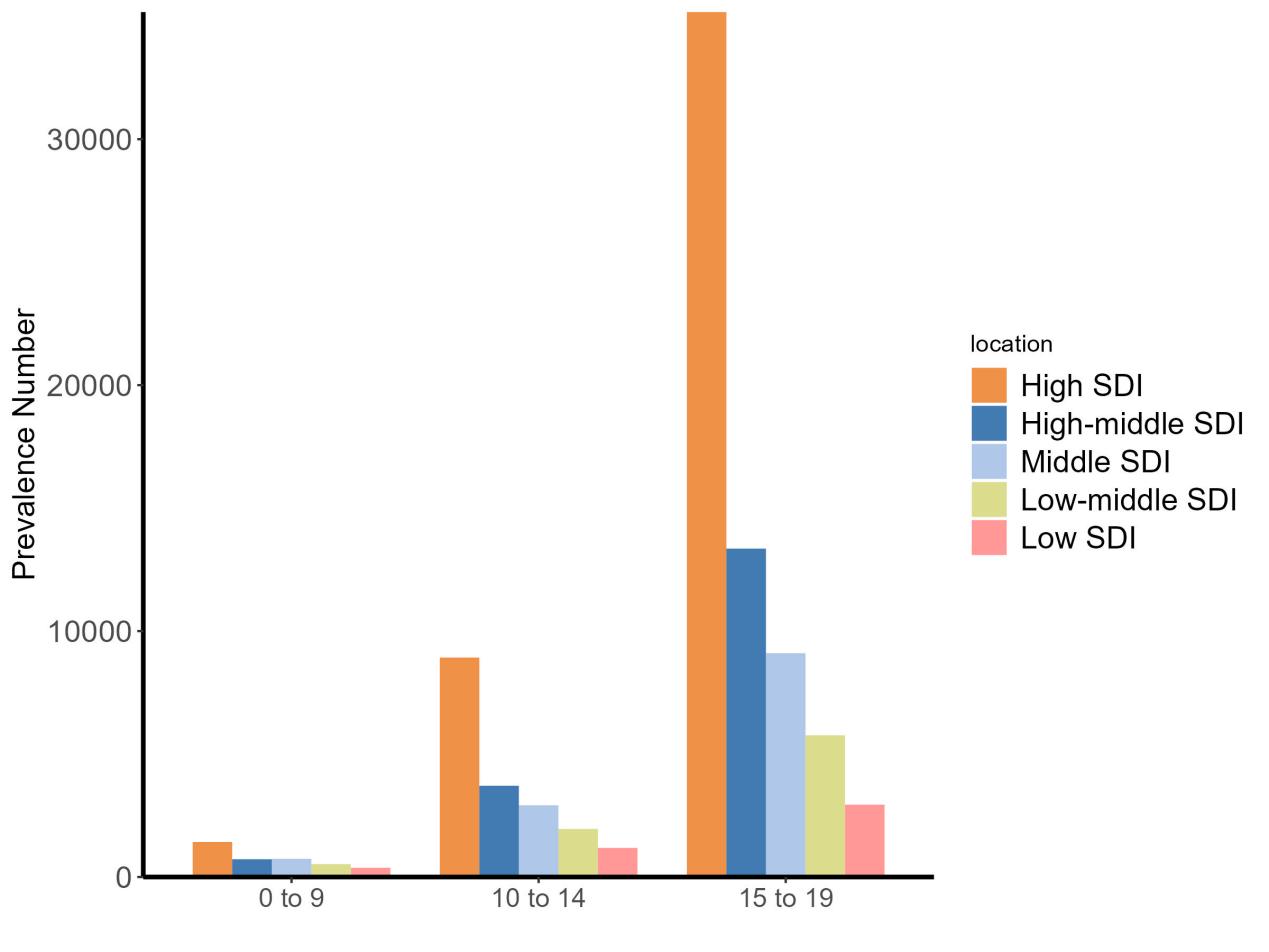


Figure S5 Children and adolescents prevalence cases of IBD in different age groups by SDI regions, 2019.


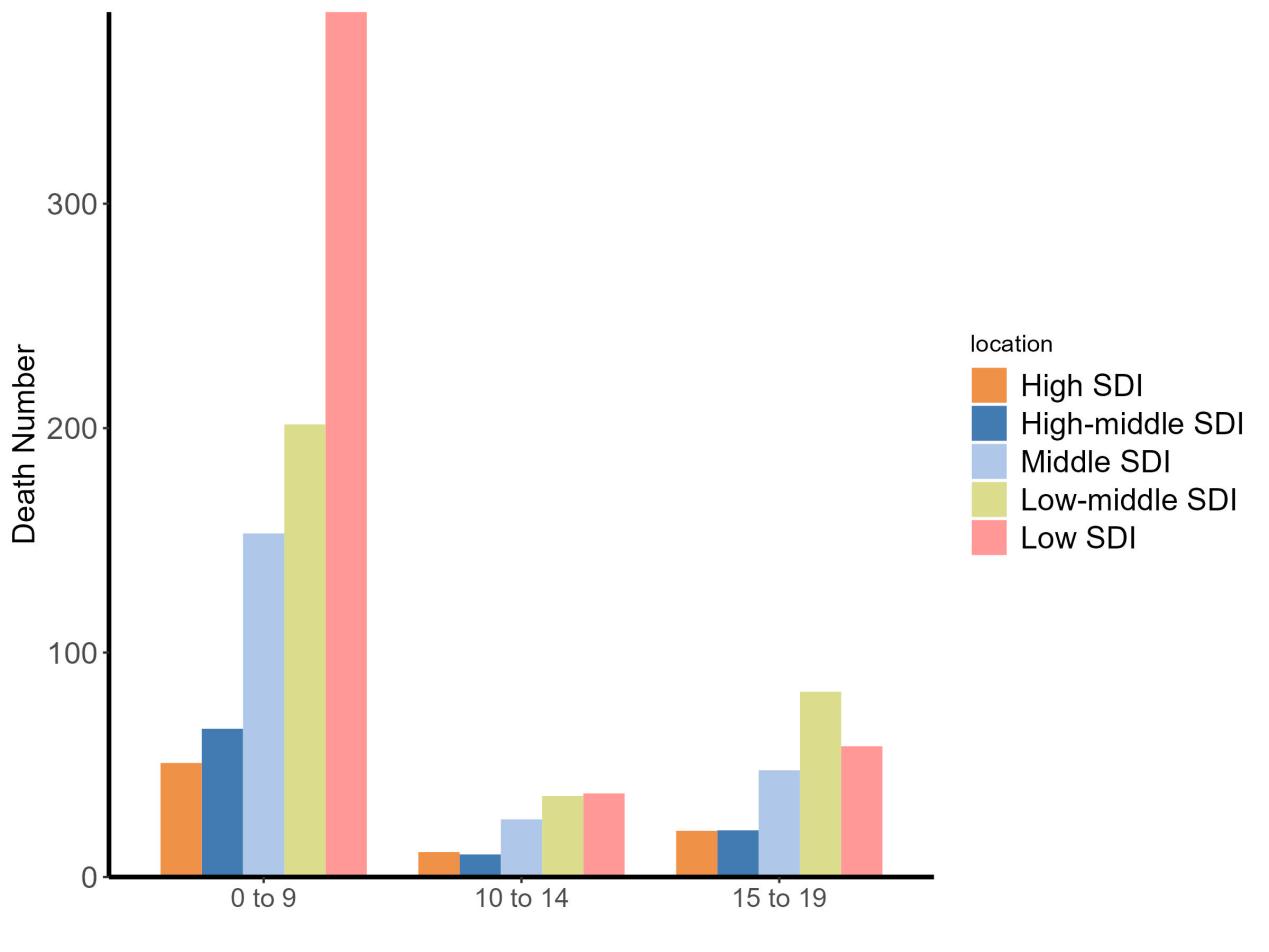


Figure S6 Children and adolescents death cases of IBD in different age groups by SDI regions, 2019.


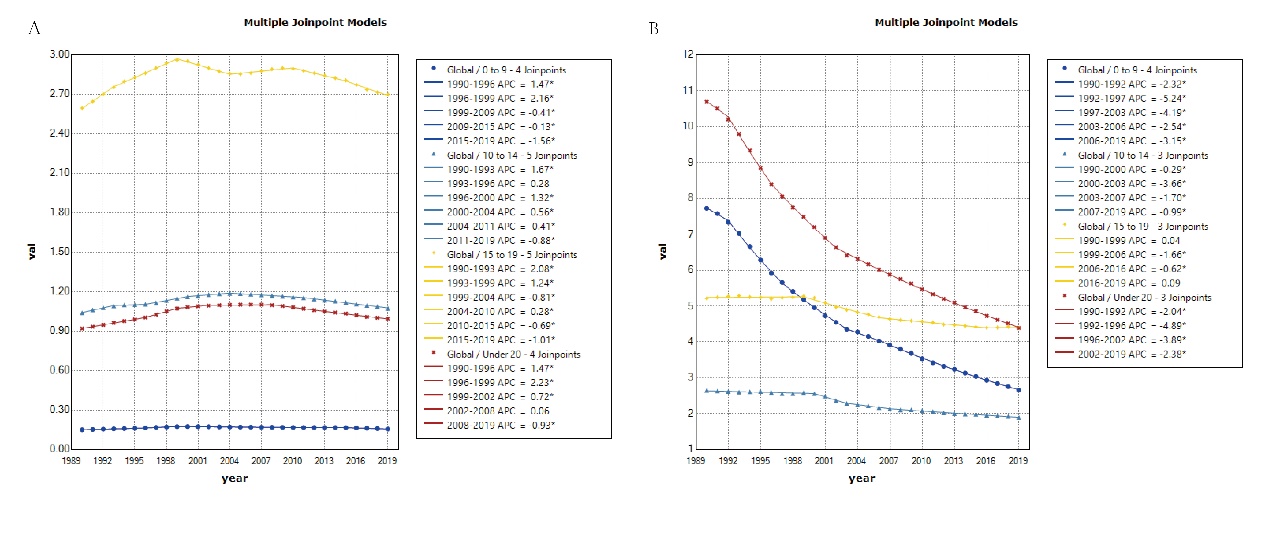


Figure S7 Joinpoint regression analysis of the early-onset IBD and non-early-onset IBD incidence and DALY rate globally from 1990 to 2019. (A) incidence rate. (B) DALY rate.


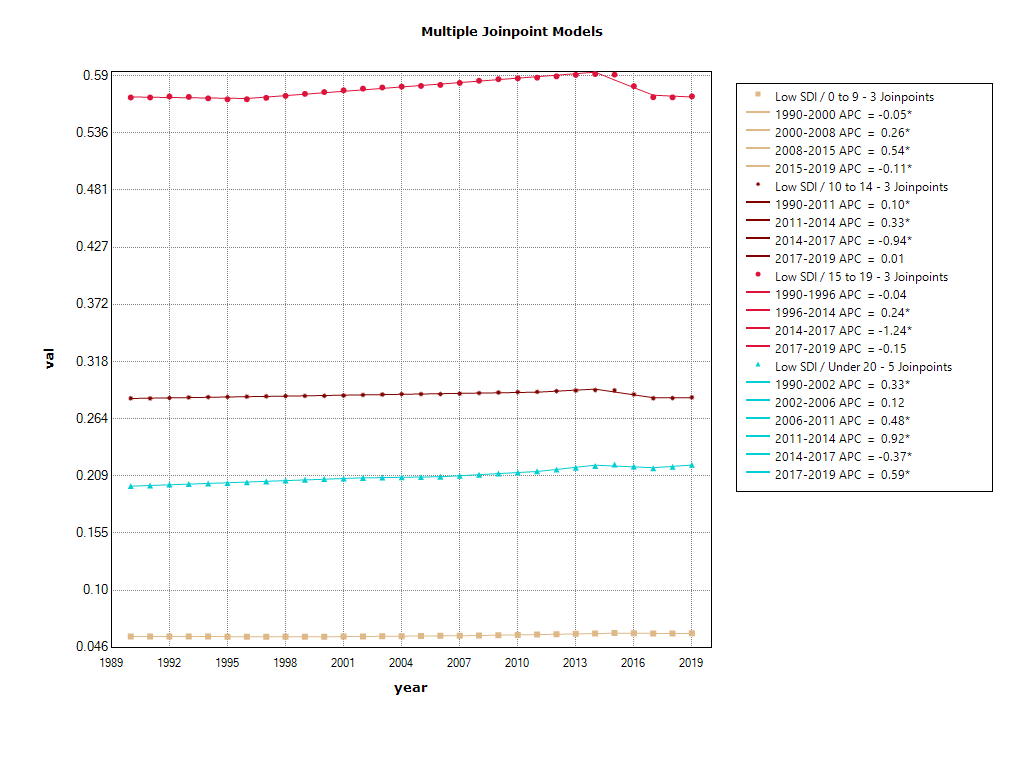


Figure S8 Joinpoint regression analysis of the early-onset IBD and non-early-onset IBD incidence rate for low SDI regions from 1990 to 2019.


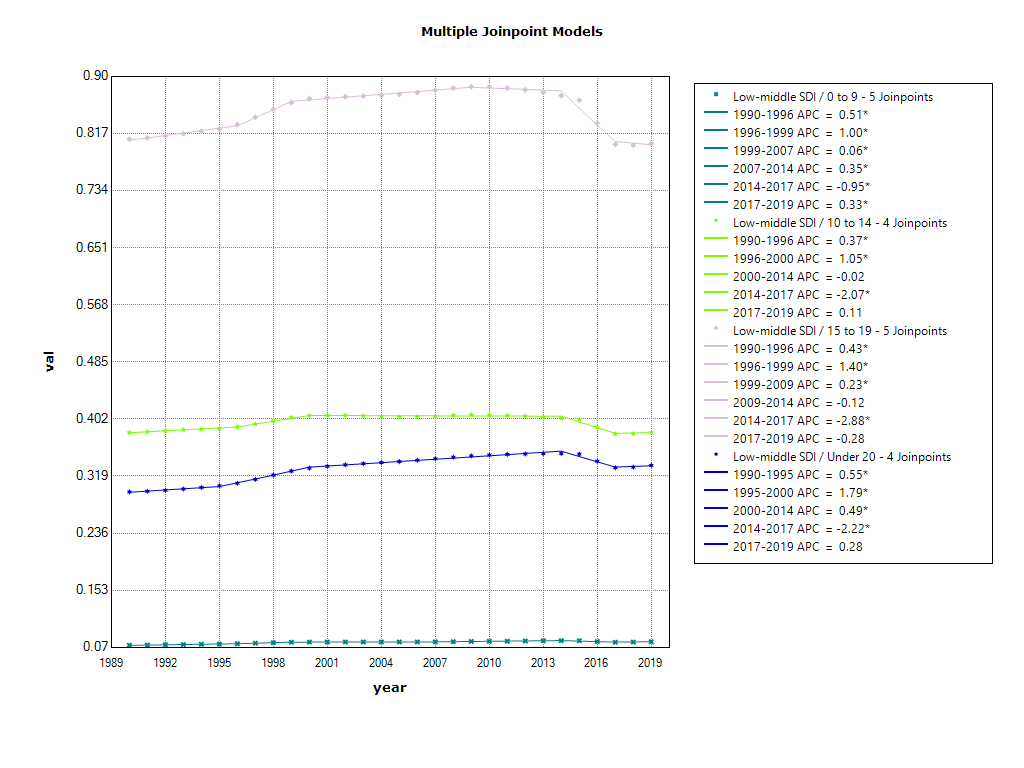


Figure S9 Joinpoint regression analysis of the early-onset IBD and non-early-onset IBD incidence rate for low-middle SDI regions from 1990 to 2019.


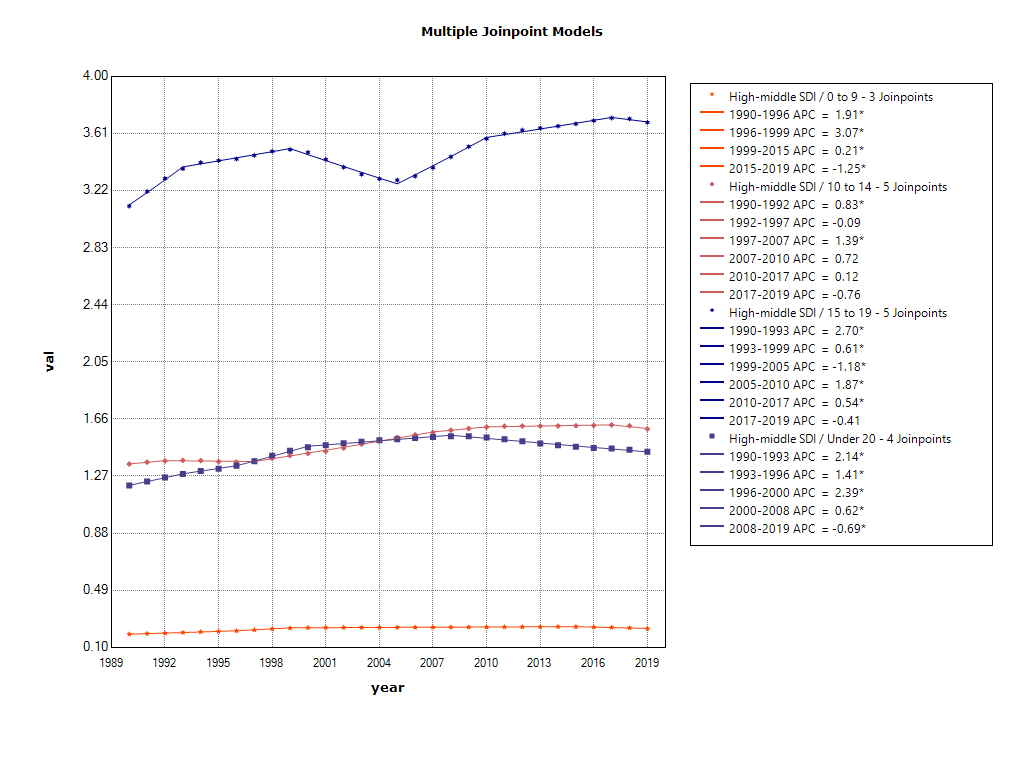


Figure S10 Joinpoint regression analysis of the early-onset IBD and non-early-onset IBD incidence rate for middle SDI regions from 1990 to 2019.


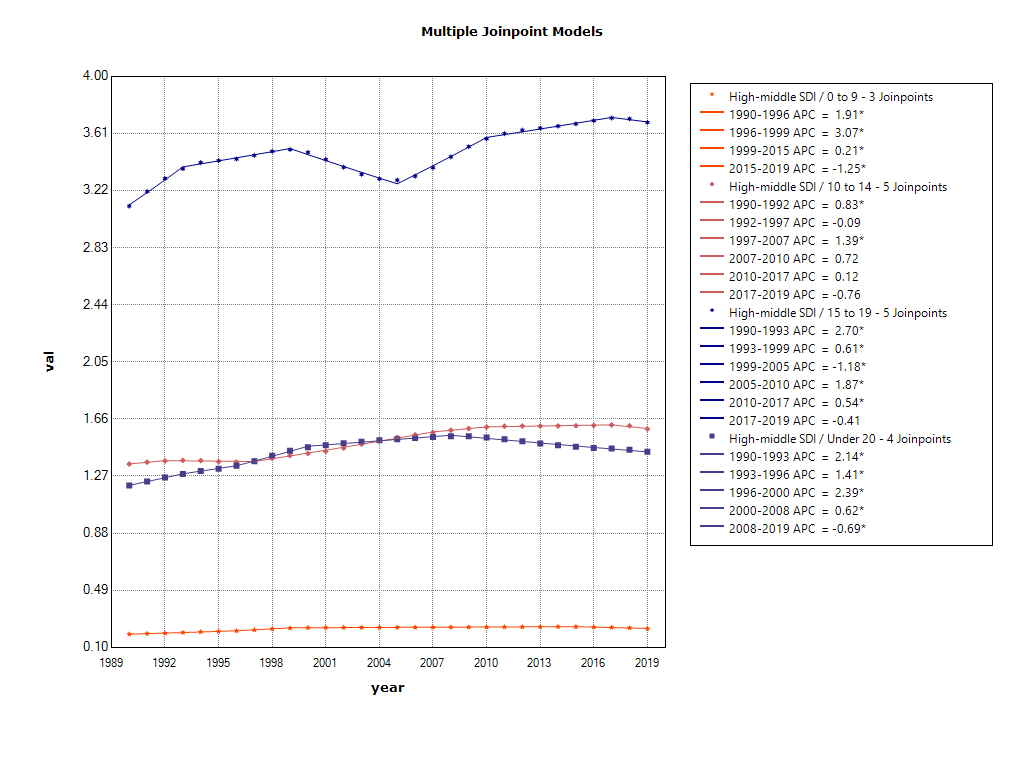


Figure S11 Joinpoint regression analysis of the early-onset IBD and non-early-onset IBD incidence rate for high-middle SDI regions from 1990 to 2019.


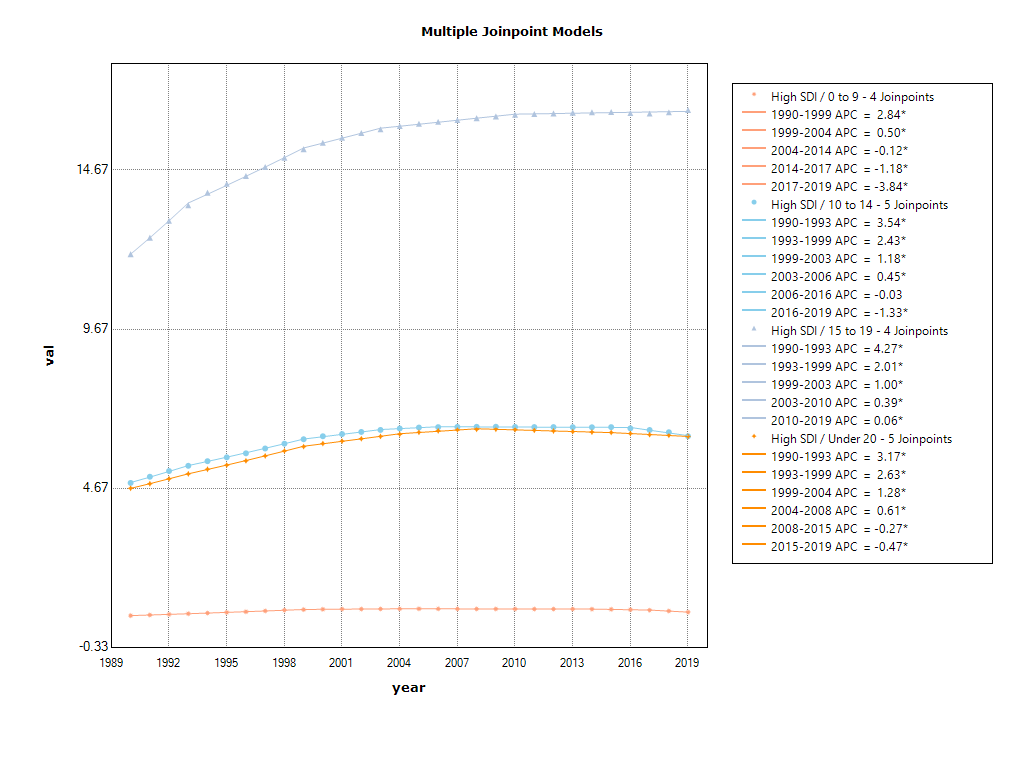


Figure S12 Joinpoint regression analysis of the early-onset IBD and non-early-onset IBD incidence rate for high SDI regions from 1990 to 2019.


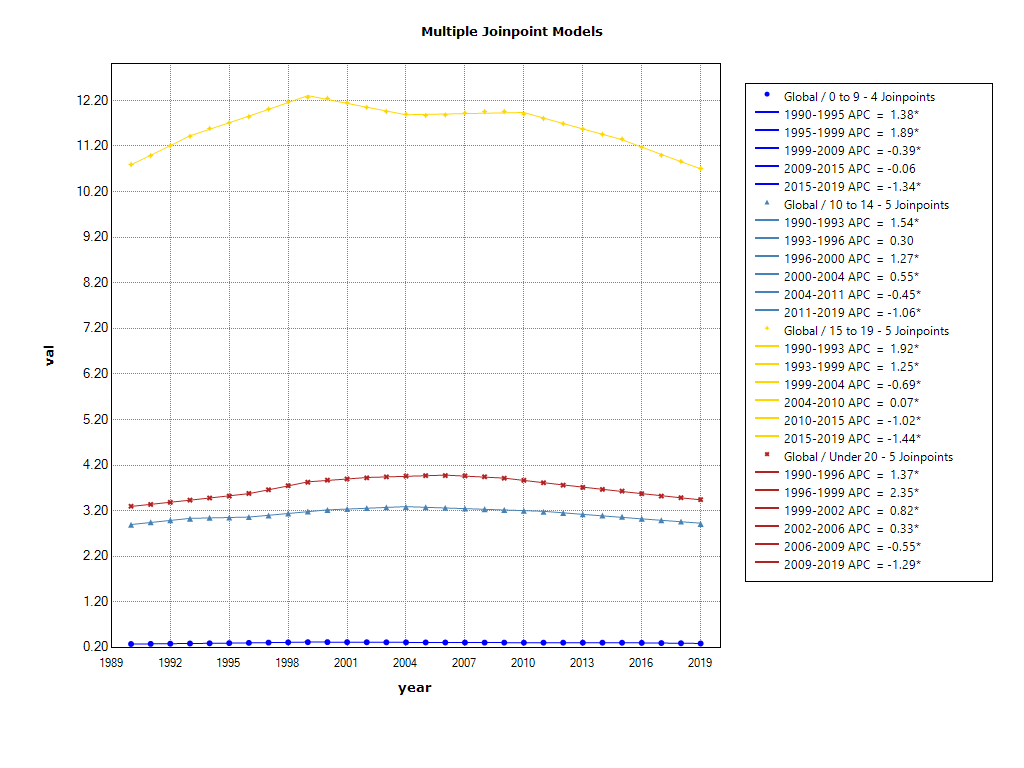


Figure S13 Joinpoint regression analysis of the early-onset IBD and non-early-onset IBD prevalence rate globally from 1990 to 2019.


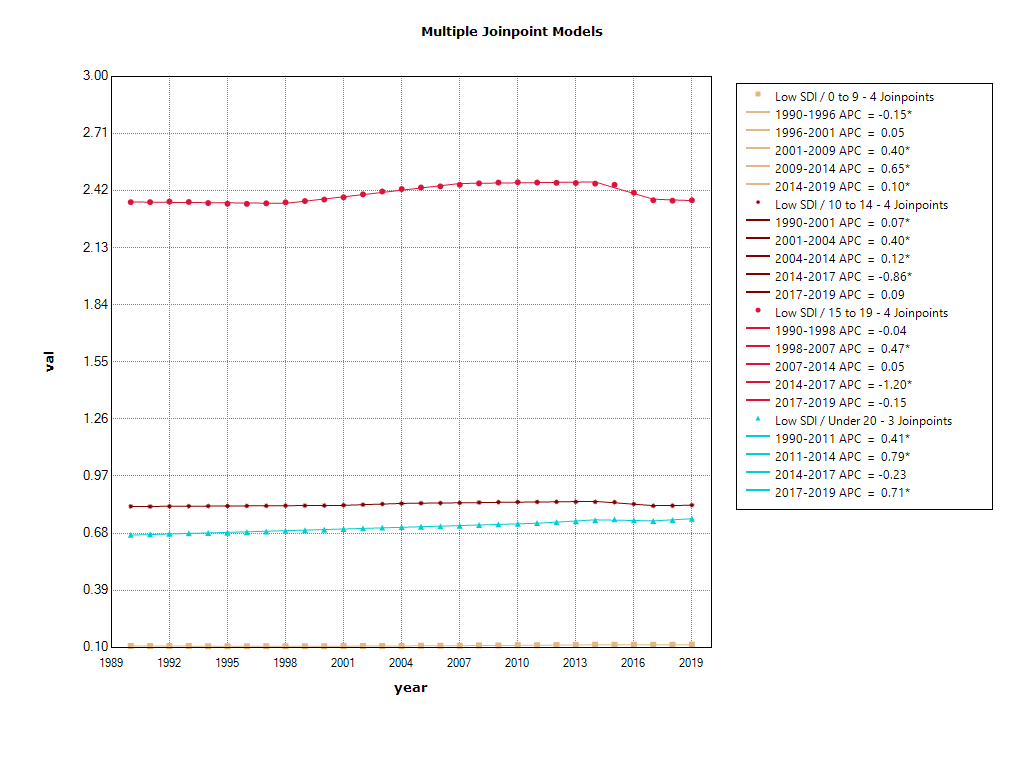


Figure S14 Joinpoint regression analysis of the early-onset IBD and non-early-onset IBD prevalence rate for low SDI regions from 1990 to 2019.


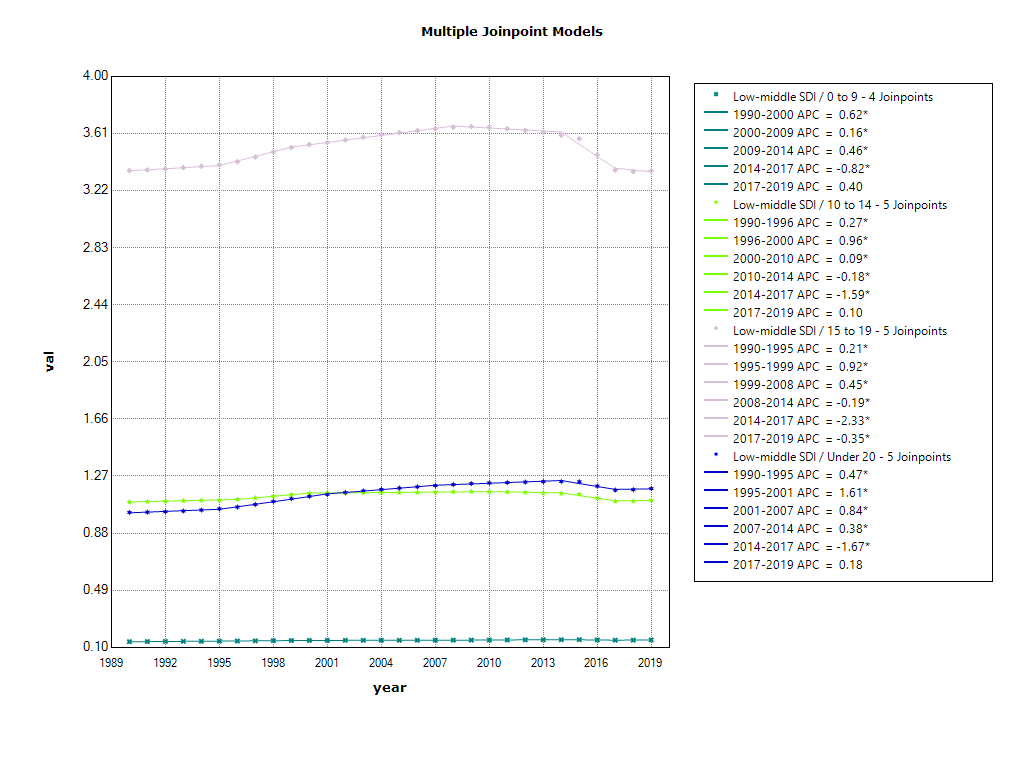


Figure S15 Joinpoint regression analysis of the early-onset IBD and non-early-onset IBD prevalence rate for low-middle SDI regions from 1990 to 2019.


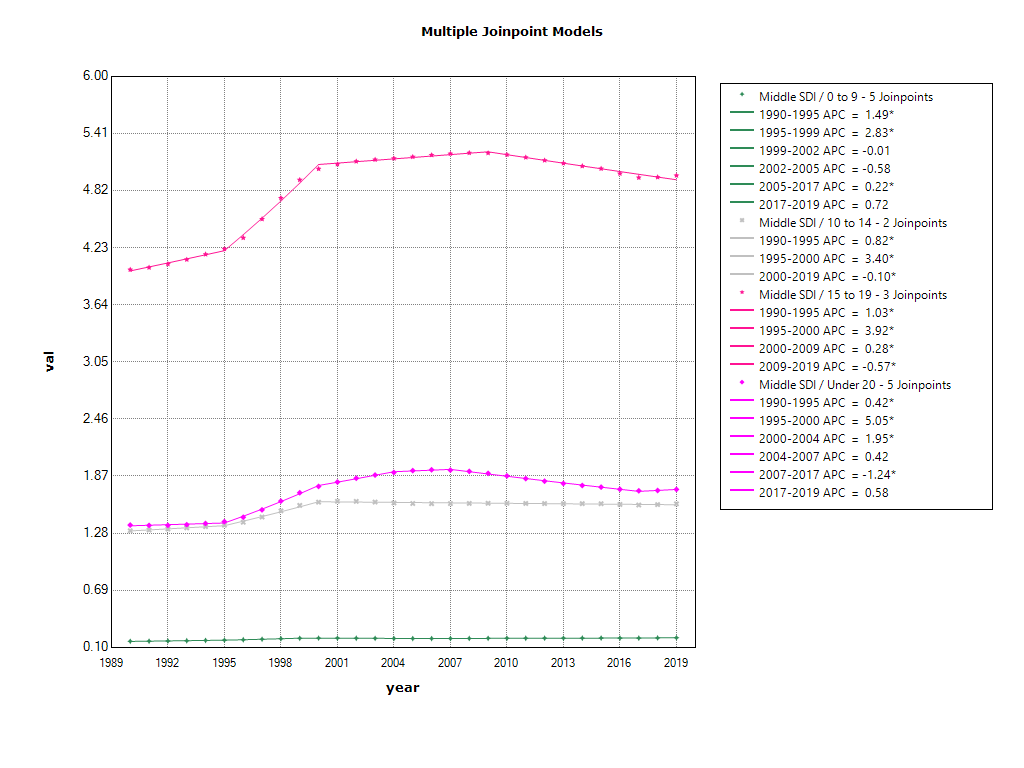


Figure S16 Joinpoint regression analysis of the early-onset IBD and non-early-onset IBD prevalence rate for middle SDI regions from 1990 to 2019.


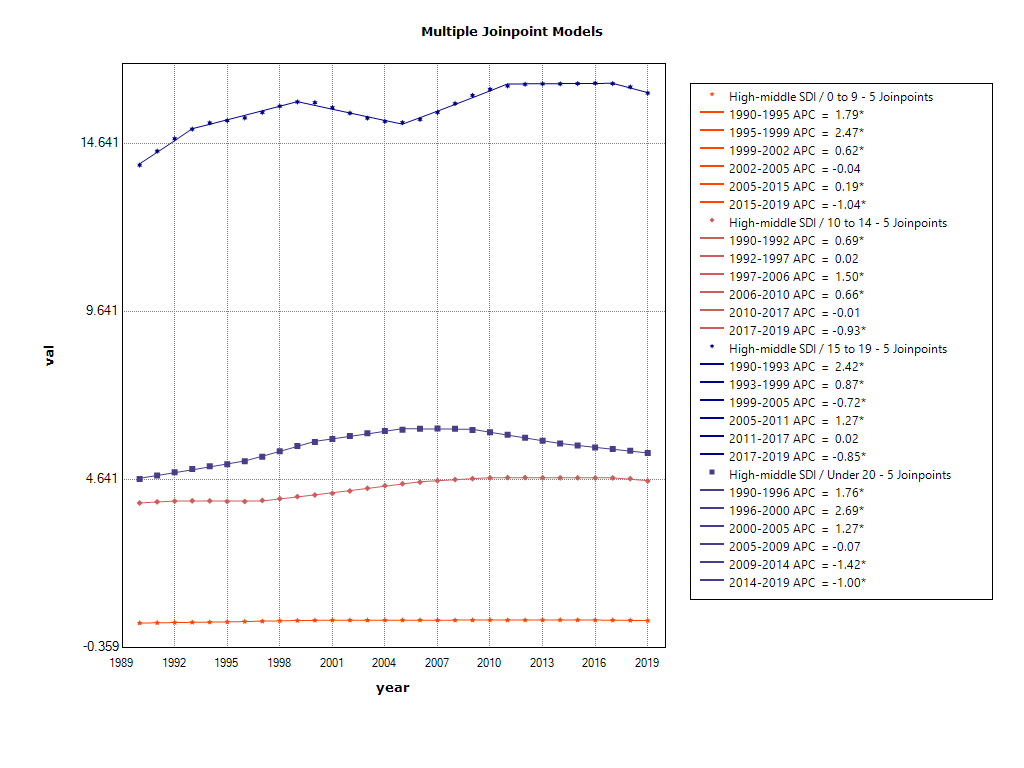


Figure S17 Joinpoint regression analysis of the early-onset IBD and non-early-onset IBD prevalence rate for high-middle SDI regions from 1990 to 2019.


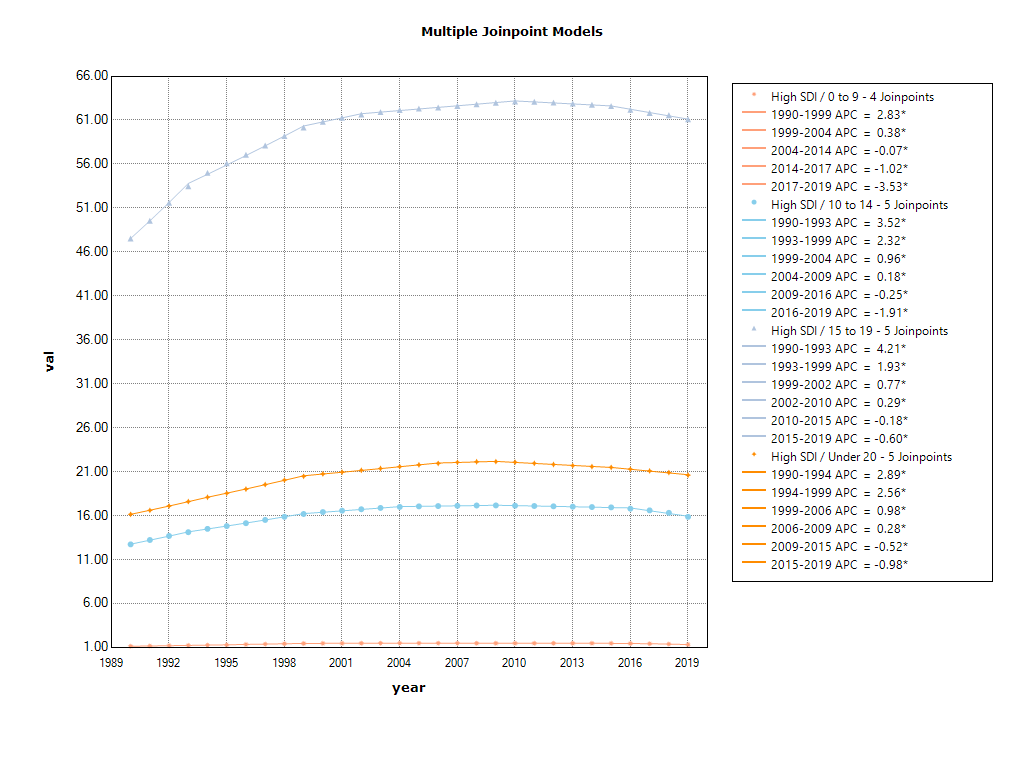


Figure S18 Joinpoint regression analysis of the early-onset IBD and non-early-onset IBD prevalence for high SDI regions from 1990 to 2019.


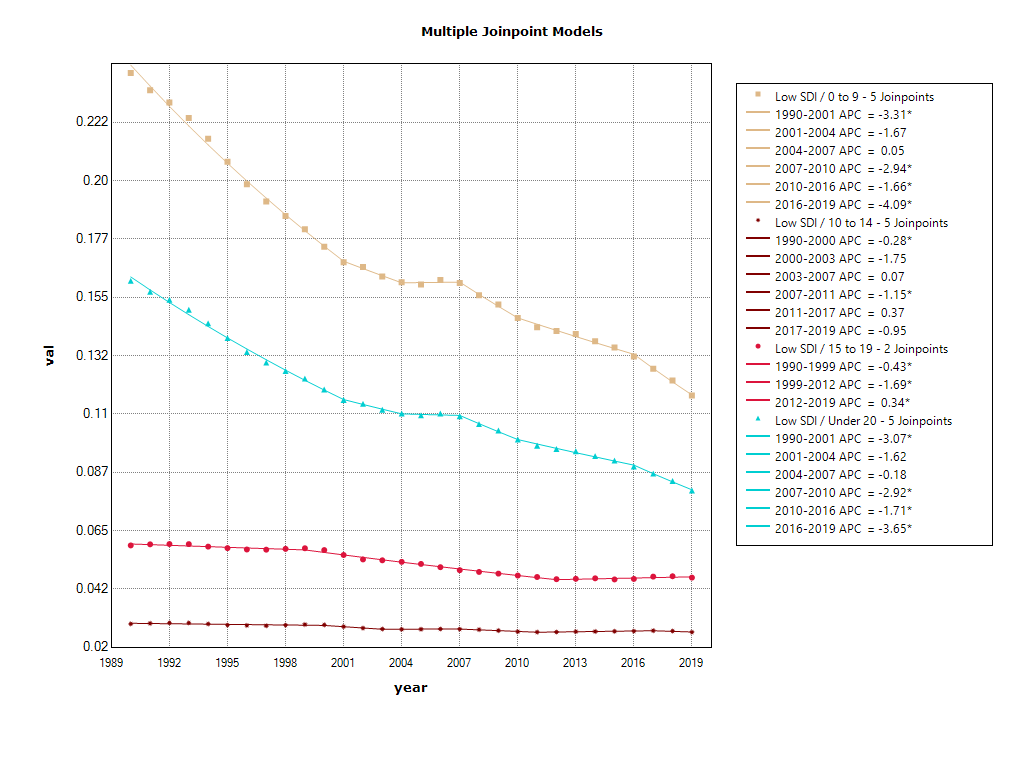


Figure S19 Joinpoint regression analysis of the early-onset IBD and non-early-onset IBD death rate for globally from 1990 to 2019.


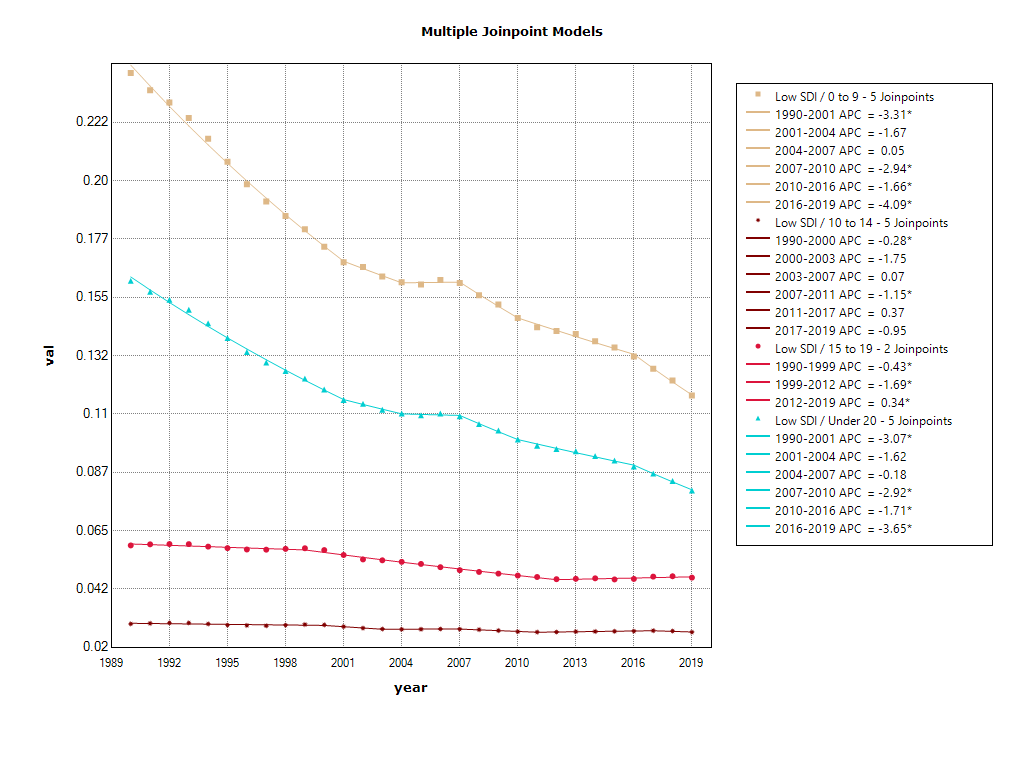


Figure S20 Joinpoint regression analysis of the early-onset IBD and non-early-onset IBD death rate for low SDI regions from 1990 to 2019.


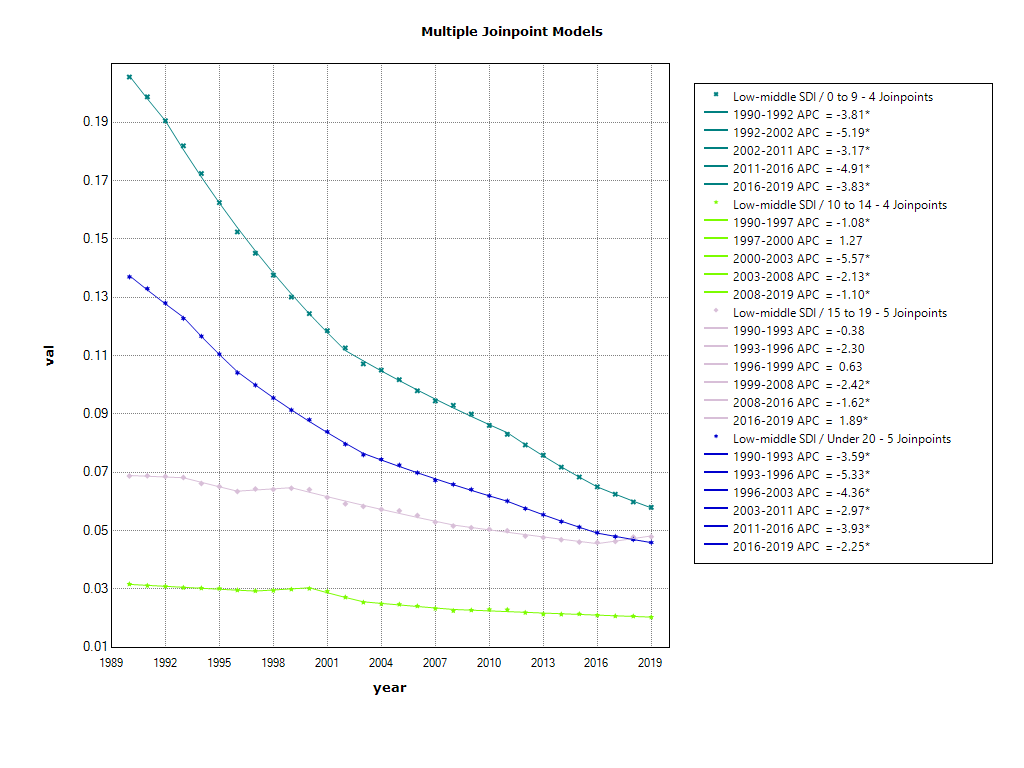


Figure S21 Joinpoint regression analysis of the early-onset IBD and non-early-onset IBD death rate for low-middle SDI regions from 1990 to 2019.


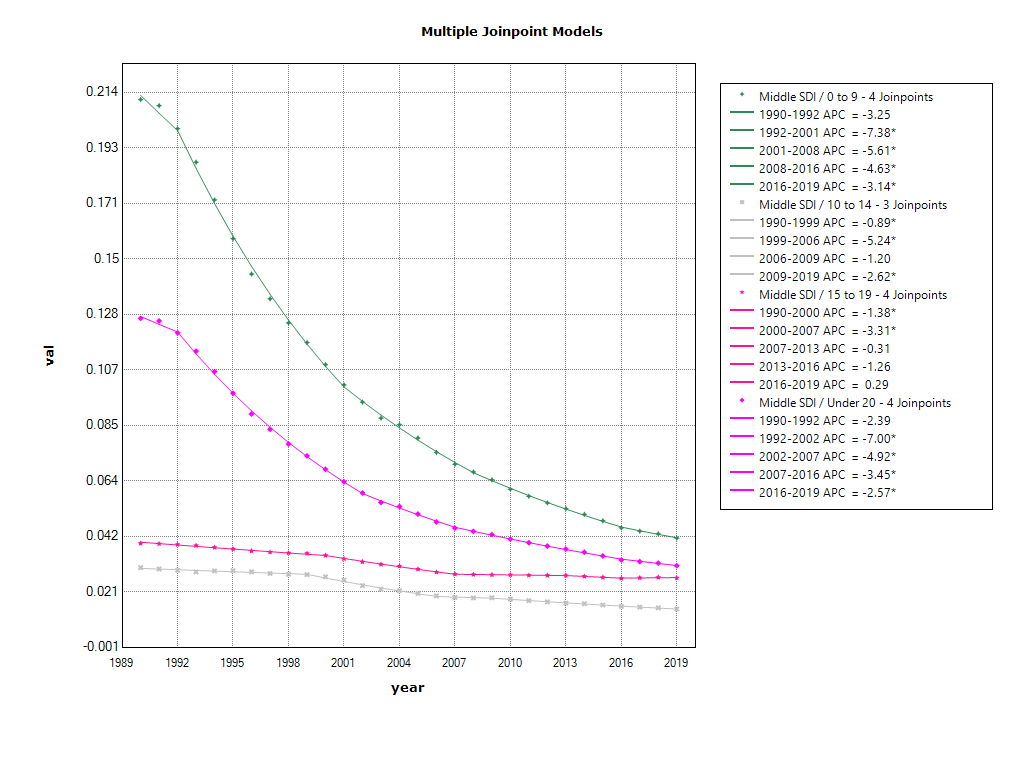


Figure S22 Joinpoint regression analysis of the early-onset IBD and non-early-onset IBD death rate for middle SDI regions from 1990 to 2019.


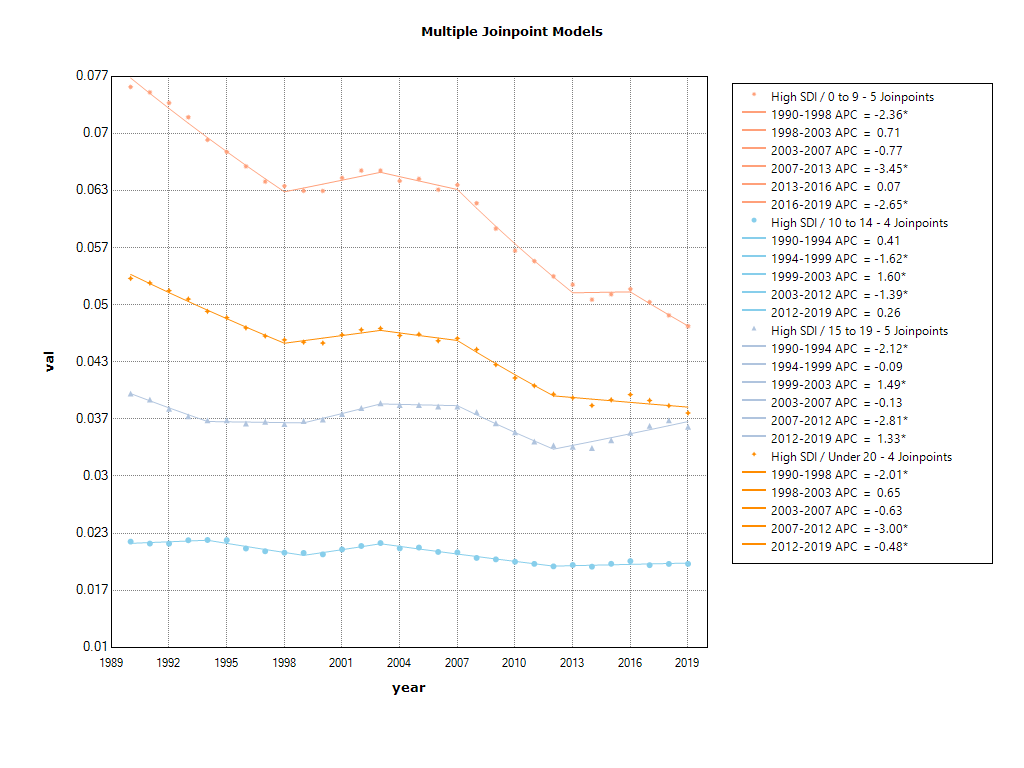


Figure S23 Joinpoint regression analysis of the early-onset IBD and non-early-onset IBD death rate for high-middle SDI regions from 1990 to 2019.


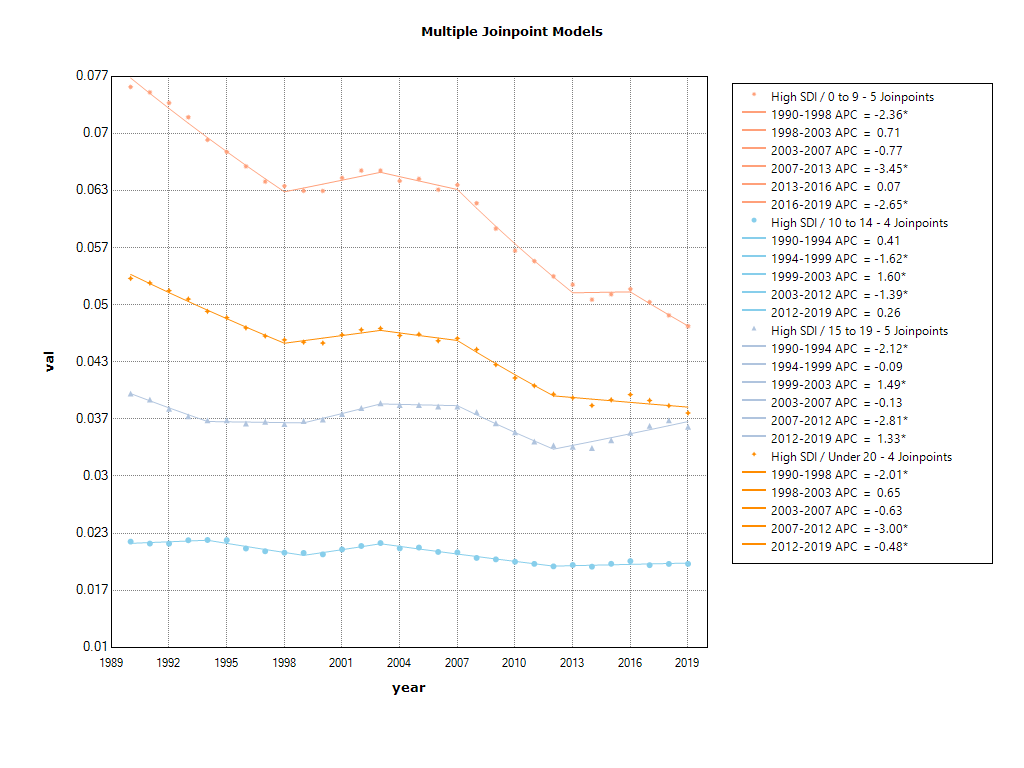


Figure S24 Joinpoint regression analysis of the early-onset IBD and non-early-onset IBD death rate for high SDI regions from 1990 to 2019.


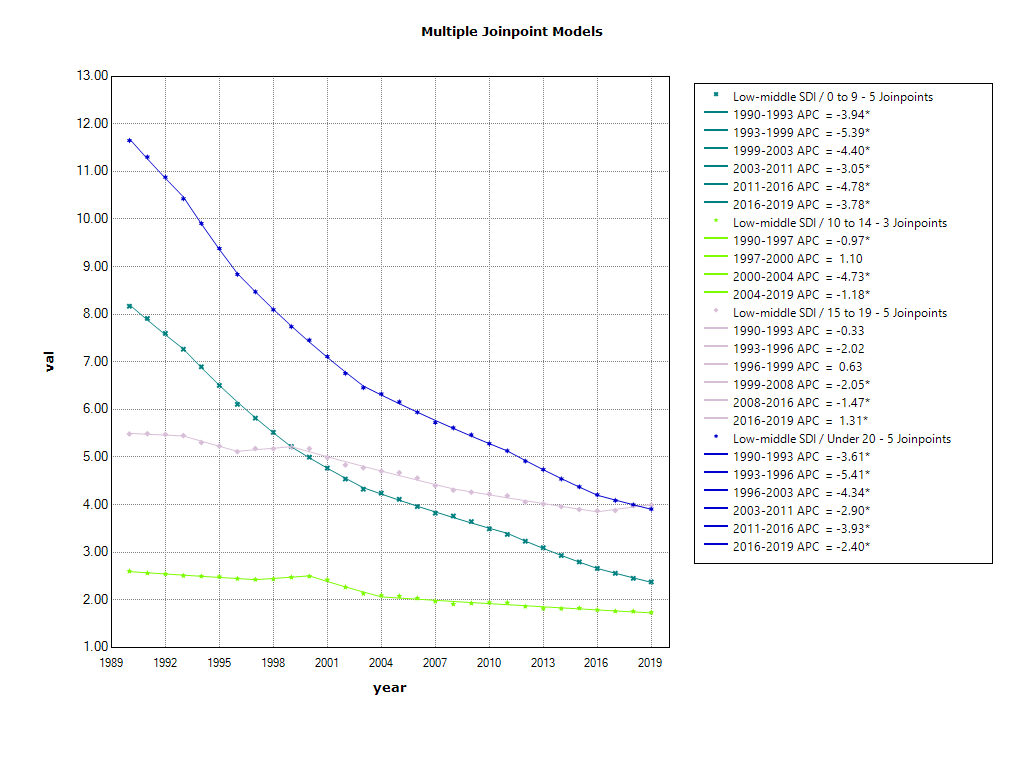


Figure S25 Joinpoint regression analysis of the early-onset IBD and non-early-onset IBD DALY rate for low SDI regions from 1990 to 2019.


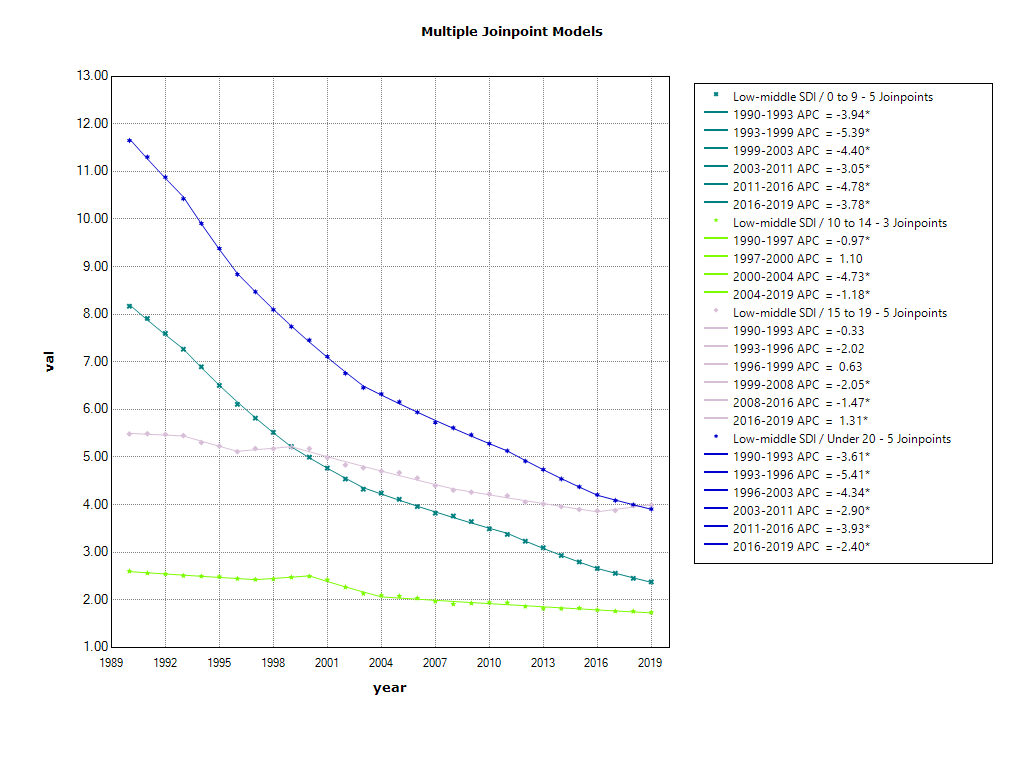


Figure S26 Joinpoint regression analysis of the early-onset IBD and non-early-onset IBD DALY rate for low-middle SDI regions from 1990 to 2019.


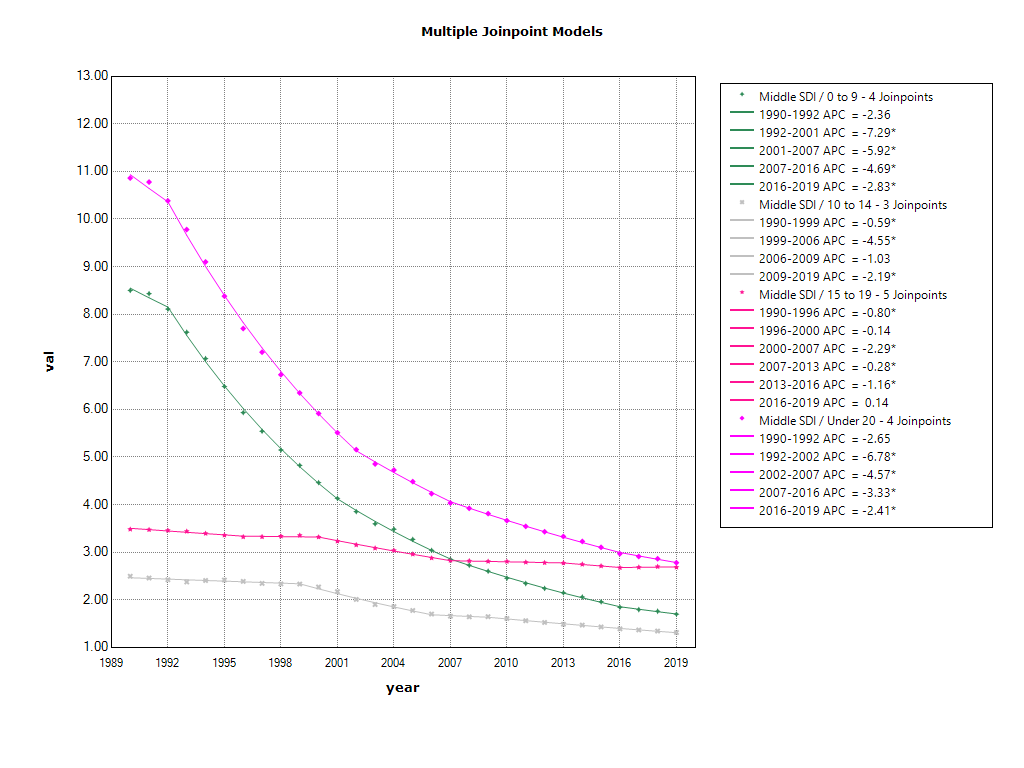


Figure S27 Joinpoint regression analysis of the early-onset IBD and non-early-onset IBD DALY rate for middle SDI regions from 1990 to 2019.


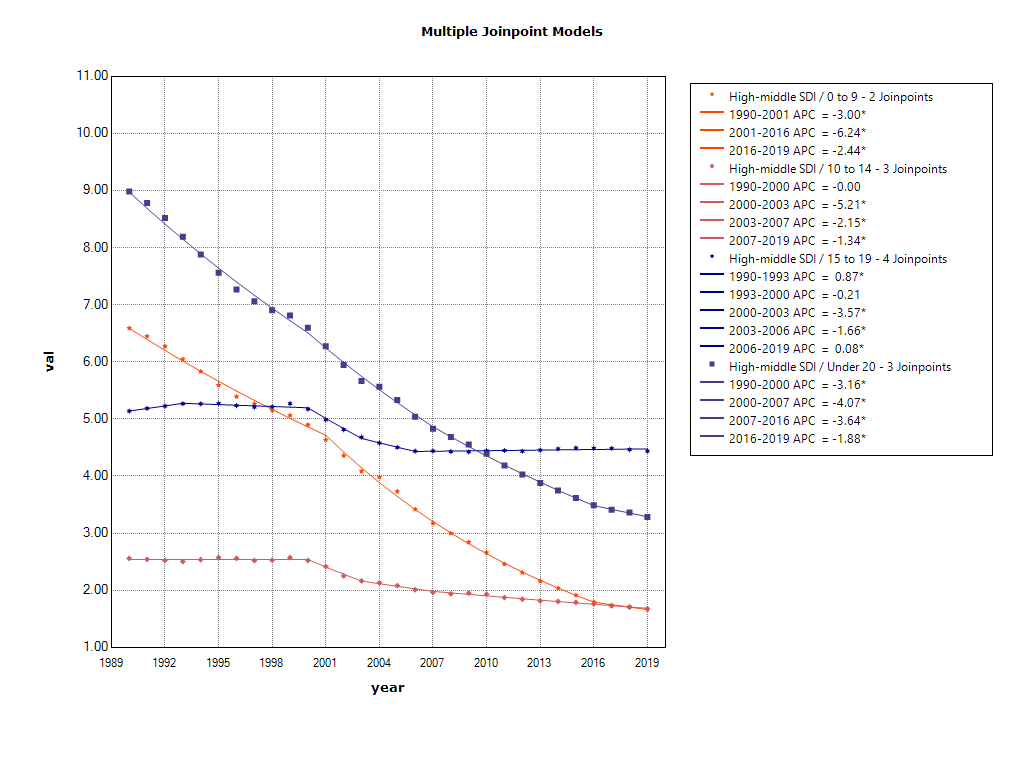


Figure S28 Joinpoint regression analysis of the early-onset IBD and non-early-onset IBD DALY rate for high-middle SDI regions from 1990 to 2019.


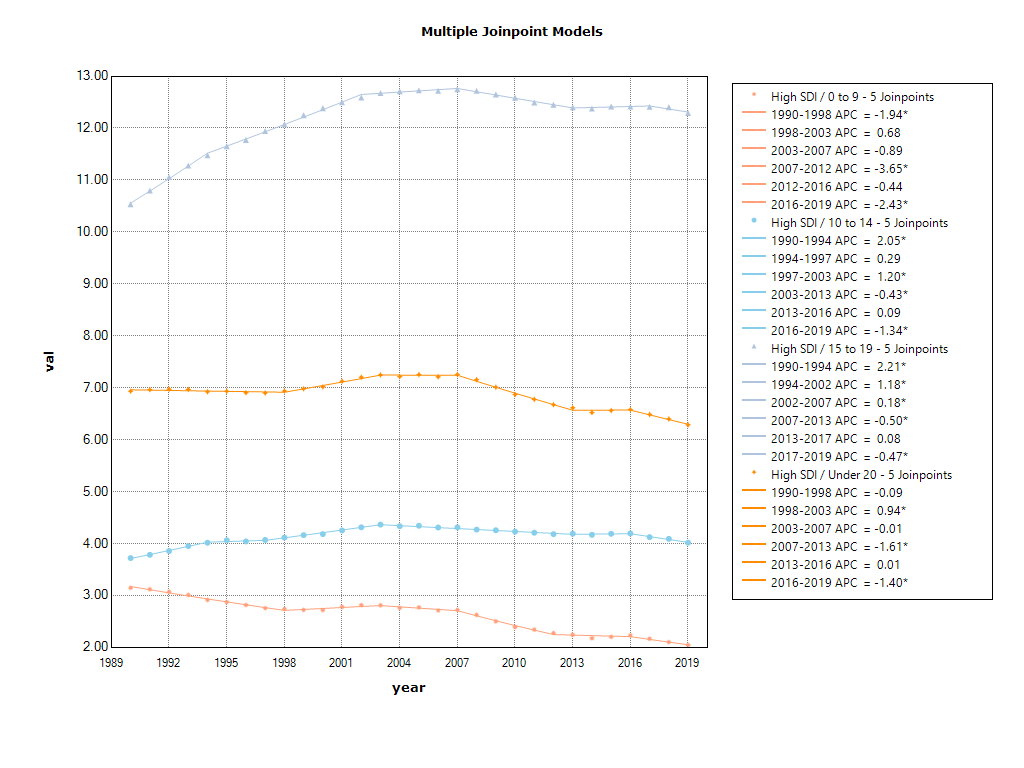


Figure S29 Joinpoint regression analysis of the early-onset IBD and non-early-onset IBD DALY rate for high SDI regions from 1990 to 2019.
